# Different genome stability proteins underpin primed and naïve adaptation in *E. coli* CRISPR-Cas immunity

**DOI:** 10.1093/nar/gkv1213

**Published:** 2015-11-17

**Authors:** Ivana Ivančić-Baće, Simon D Cass, Stephen J Wearne, Edward L Bolt

**Affiliations:** 1 Faculty of Science, Department of Molecular Biology, University of Zagreb, Horvatovac 102a, Zagreb, Croatia; 2 School of Life Sciences, Queen's Medical Centre, University of Nottingham, NG72UH, UK

## Abstract

CRISPR-Cas is a prokaryotic immune system built from capture and integration of invader DNA into CRISPR (Clustered Regularly Interspaced Short Palindromic Repeats) loci, termed ‘Adaptation’, which is dependent on Cas1 and Cas2 proteins. In *Escherichia coli*, Cascade-Cas3 degrades invader DNA to effect immunity, termed ‘Interference’. Adaptation can interact with interference (‘primed’), or is independent of it (‘naïve’). We demonstrate that primed adaptation requires the RecG helicase and PriA protein to be present. Genetic analysis of mutant phenotypes suggests that RecG is needed to dissipate R-loops at blocked replication forks. Additionally, we identify that DNA polymerase I is important for both primed and naive adaptation, and that RecB is needed for naïve adaptation. Purified Cas1-Cas2 protein shows specificity for binding to and nicking forked DNA within single strand gaps, and collapsing forks into DNA duplexes. The data suggest that different genome stability systems interact with primed or naïve adaptation when responding to blocked or collapsed invader DNA replication. In this model, RecG and Cas3 proteins respond to invader DNA replication forks that are blocked by Cascade interference, enabling DNA capture. RecBCD targets DNA ends at collapsed forks, enabling DNA capture without interference. DNA polymerase I is proposed to fill DNA gaps during spacer integration.

## INTRODUCTION

CRISPR-Cas are adaptive immune systems in prokaryotes that act against invasive genetic elements (e.g. phages and plasmids) (1). Immunity is based on a CRISPR (Clustered Regularly Interspaced Short Palindromic Repeats (2,3)) locus that comprises numerous repeat DNA sequences alternating with ‘spacer’ DNA sequences derived from an invader. Cas (CRISPR-associated) proteins catalytically process CRISPR DNA and RNA to bring about immunity through targeting and destruction of invader nucleic acids. Building of the CRISPR immune system requires Cas1 and Cas2 proteins, and is likely to occur in two major events; capture of DNA fragments (‘protospacers’) from an invader, and integration of protospacers into a CRISPR locus as a new spacer. Spacer integration is accompanied by synthesis of a new repeat by an unknown factor. These processes are called CRISPR ‘Adaptation’ or ‘Acquisition’ (1,4–9).

Transcription of a CRISPR locus yields ‘pre-crRNA’ that is cleaved to ‘crRNA’ within repeat sequences and assembled into ribonucleoprotein complexes (Cascades (10), Cas9 (11), CMR (12) and CSM (13)). Each crRNA comprises a spacer sequence that is targeted by these complexes to homologous invader nucleic acids, triggering their degradation. These processes are termed ‘interference’. Interference complexes show mechanistic and/or structural differences, reflected by classification of CRISPR-Cas systems into two major Classes that comprise five Types (I–V), with further division into 16 sub-Types (14). In Type I CRISPR-Cas systems, which include *Escherichia coli*, interference with invader DNA is catalyzed by the ‘Cascade’ ribonucleoprotein protein complex and the Cas3 translocase-nuclease (10). Cascade catalyses base pairing of crRNA to the double stranded DNA protospacer, producing a structure called an R-loop (15,16). Structural analyses of Cascade complexes have revealed details of crRNA nucleoprotein filament formation and their targeting to DNA (15,17–21). Cascade initiates interference by binding to negatively supercoiled DNA at sequences called ‘Protospacer Adjacent Motifs’ (PAMs) located in invader DNA (22–24,25,26). CRISPR loci lack PAMs, providing a mechanism to prevent self-destruction by interference. In *E. coli* Cascade, sub-unit Cse1 binds to a PAM (8,19,27,28), beginning R-loop formation between the crRNA and protospacer in an eight-nucleotide seed that is extended over 30–33 nucleotides to conformationally lock Cascade (28–30). ‘Escape’ mutations or polymorphisms in PAM or protospacer DNA cause mismatches in crRNA–DNA that alter the disposition of Cascade reducing the effectiveness of interference, resulting in incomplete immunity (30–32).

Intriguing interplay between adaptation and interference has been observed, when escape mutations that influence Cascade binding to invader DNA also stimulate Cas1-Cas2 catalyzed adaptation (4,6,32–34). This is called ‘primed’ adaptation and relies on Cascade binding to a non-optimal PAM or with bound crRNA from a pre-existing spacer that imperfectly matches a protospacer. Therefore, primed adaptation can re-establish immunity against an invader that would otherwise have acquired resistance. The genetic requirements for primed adaptation are defined as *cas1, cas2, cas3, cascade* and a sub-optimal PAM or a spacer that imperfectly matches a protospacer target (32). Adaptation in the absence of interference, termed ‘naïve adaptation’ (35), generates immunity against an invader that has not been previously encountered. The ability of Cas1 and Cas2 to catalyze naïve adaptation independently of Cascade has been demonstrated *in vivo* (5,36) and using purified Cas1 and Cas2 proteins (37).

In *E. coli*, adaptation requires catalytic activity from Cas1 in complex with Cas2 forming an oligomer of two or four Cas1 monomers (36). The integration stage of adaptation, generating a new spacer-repeat pair within a CRISPR locus, proceeds by Cas1 nicking the first repeat giving 5′ DNA ends that are joined to 3′ protospacer ends *via* transesterification reactions (36–38). Integration targets CRISPR repeat DNA that may form structures influenced by DNA supercoiling or other factors (37). Less is known about how protospacer DNA capture occurs prior to integration, although replication forks at *ter* sites in *E. coli* provide a major source of new spacer DNA in naïve adaptation (39). The same analysis also highlighted a fascinating role for the RecBCD complex in naïve adaptation, providing a mechanism for DNA capture that could specify invader DNA rather than host DNA.

We investigated requirements for *E. coli* host genomic stability proteins during adaptation, comparing naïve and primed adaptation because of their potential differences owing to the absence or presence of Cascade interference. Genetic analysis demonstrated that DNA polymerase I, RecG and PriA facilitate primed adaptation. DNA polymerase I and RecB were needed for naïve adaptation, but RecG was not needed. Genetic analysis of *recG* and *priA* in primed adaptation gave phenotypes corresponding to known roles of RecG at blocked replication forks, and indicated that RecG is required to remove R-loop complexes. We analyzed activities of purified Cas1 and Cas2 proteins at low concentrations (0–25 nM), and observed strong preference for binding and catalysis targeted to single stranded DNA gaps in fork substrates. A model is presented suggesting new roles for genome stability enzymes that underpin CRISPR immunity.

## MATERIALS AND METHODS

### Strains, plasmids and reagents

Gene deletion strains are listed in Supplementary Table S1, and plasmids for genetic analysis and protein purification are listed in Supplementary Table S2. Some strains were obtained from the Coli Genetic Stock Center (CGSC) (http://cgsc.biology.yale.edu/DatabaseInfo.php) and further manipulated using P1 transductions and strain verification as described in Supplementary Material. The Δ*recG* Δ*priA* double mutant required for data shown in Figure 2 was constructed by P1 transduction of Δ*recG* into Δ*priA* cells containing pPriA300 to maintain viability. The Δ*polA* strain used is described in (40)(JJ1038) and lacks polymerase function but has improved viability because it retains the exonuclease domain.

### Phage infectivity assays for spacer acquisition

**Figure 1.**
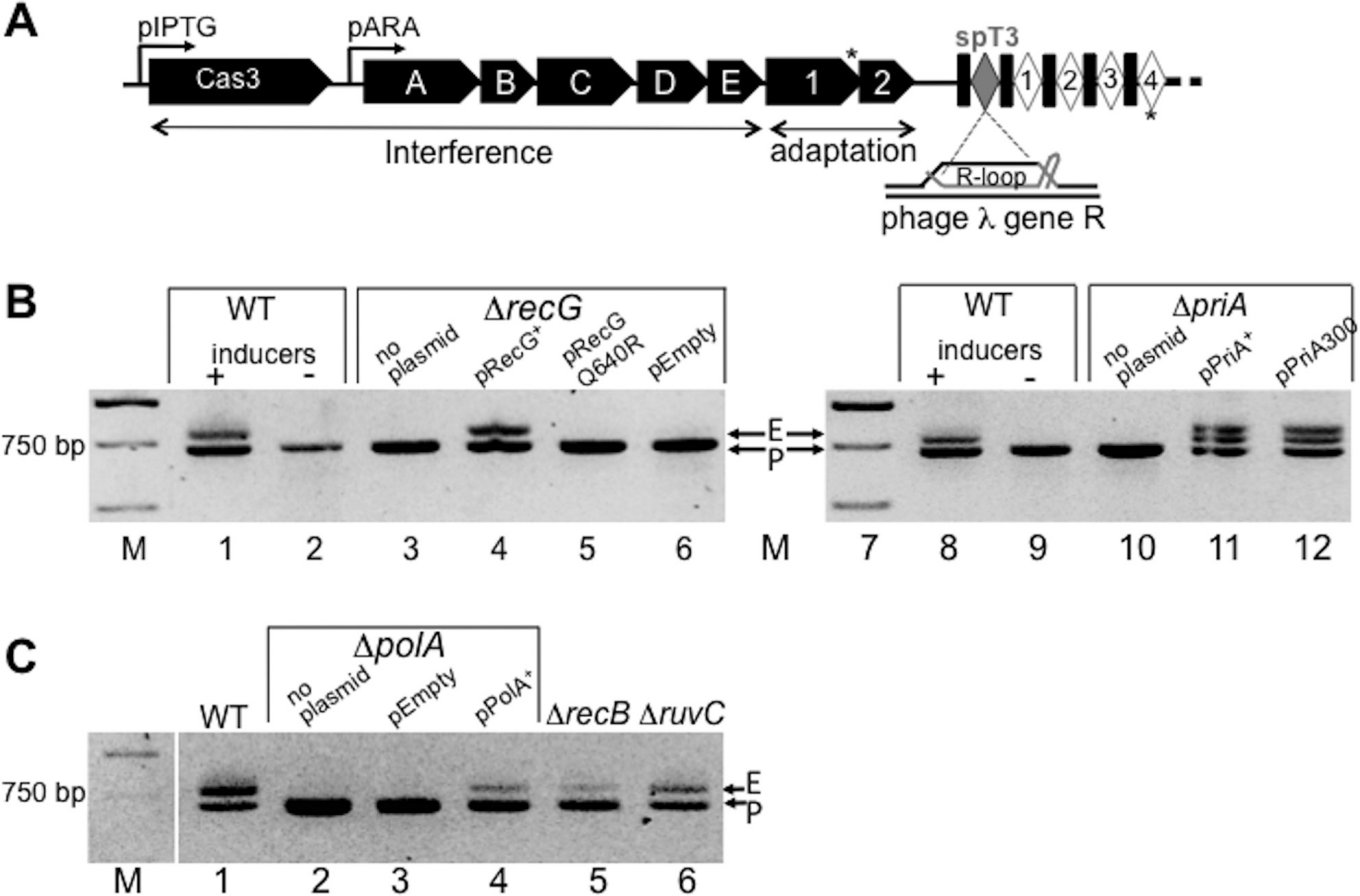
RecG, PriA and DNA polymerase I (PolA) are needed for primed adaptation. (**A**) Summary of the modified *Escherichia coli* CRISPR-Cas system used to analyze primed adaptation. Genes encoding the interference complex (*cas3*, and *casA-E ‘cascade’*), the adaptation complex (*cas1, cas2*) and the crRNA spacer T3 (spT3), which are all required for priming, were induced by arabinose (pARA) and IPTG (pIPTG). Asterisks illustrate annealing positions of primers used in PCR reactions to detect CRISPR expansion. In CRISPR, each repeat is denoted as a filled rectangle and each spacer as a diamond. (**B**) DNA gels from PCR reactions assaying for CRISPR expansion in the ‘wild type’ (wt) strain for primed adaptation (lanes 1 and 2), compared to isogenenic strains with gene deletions in *recG* or *priA* as indicated. E indicates CRISPR expansion from a 723 bp parental length, P, to 784 bp, when CRISPR-Cas was induced by addition of arabinose and IPTG (lane 1), compared to without inducers (lane 2). Plasmid expression of missing proteins, or their active site mutants, was used as indicated to determine if primed adaptation could be restored in each case. Agarose gels were stained using ethidium bromide and are displayed in reverse contrast. (**C**) Agarose gel as in (B), showing loss of primed adaptation from deleting *polA* (lane 2), and its complementation by plasmid *polA* (pPolA^+^, lane 4), compared to empty plasmid vector (lane 3) and to *recB* and *ruvC* gene deletions (lanes 5 and 6).

The strain used for primed adaptation (Figure 1A) contained the CRISPR-Cas genetic elements defined as necessary for priming (32). The engineered spacer (spT3) was to target an essential gene of a virulent lambda phage (λ *vir*) (41). Primed adaptation was assayed in *E. coli* strain IIB969 (Figure 1A) and its derivatives. Overnight cultures of appropriate strains were inoculated into LB containing inducers IPTG (1.0 mM) and arabinose (0.2% w/v) as indicated. At optical density (OD_600_) of 0.3, λ *vir* was added to a multiplicity of infection (MOI) of 1.0 followed by phage adsorption for 20 min. Cells were then diluted 1:10 into fresh LB containing inducers as required and growth was continued for 12–16 h. These infectivity assays were repeated at least three times, to monitor spacer acquisition by PCR, using primers annealing to CRISPR positions annotated by asterisks in Figure 1A, detailed in Supplementary Methods. Template DNA was derived from either bacterial culture lysed by boiling in water, or from purified genomic DNA extracted from 1 ml of bacterial culture using a kit (GeneJET, Thermo Scientific). For each different culture the OD_600_ was measured and cultures were diluted to be at equal turbidity prior to isolating DNA. Typical OD_600_ values observed for deletion strain cultures during these assays are given in Supplementary Table S5B. Individual survivor colonies obtained from plated cell cultures were picked for DNA sequencing corresponding to newly acquired spacer, as shown in Supplementary Table S3. PCR products were analyzed on 2.0% agarose Tris-acetate-EDTA (TAE) gels. If no spacer acquisition was detected in CRISPR-1, the same PCR method was used to monitor CRISPR-2 for expansion, using primer pairs listed in Supplementary Materials. If there was still no detectable spacer acquisition, infectivity assays were repeated with further rounds of infectivity using the same method as described above.

**Figure 2.**
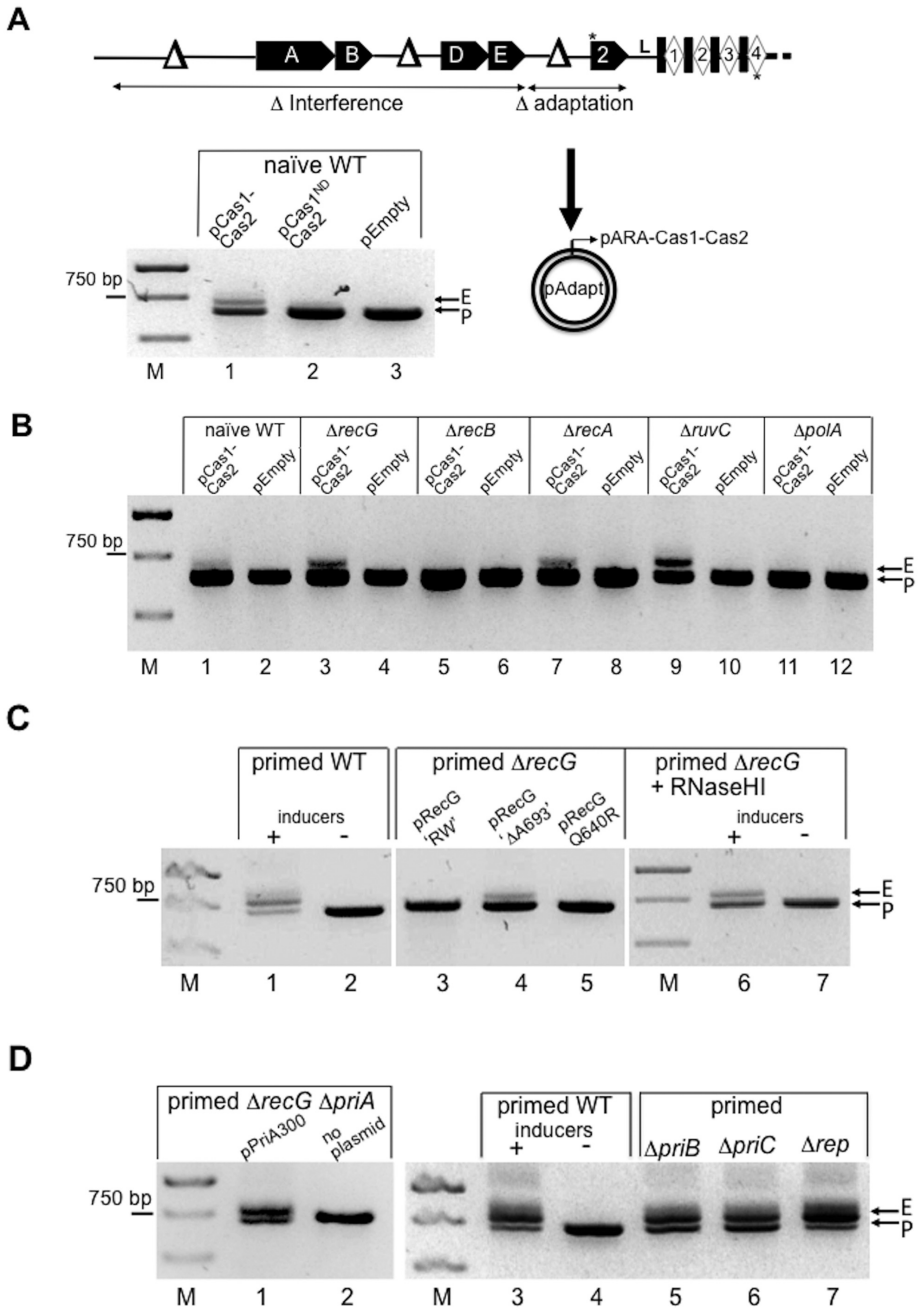
(**A**) A summary of the modified *E. coli* CRISPR-Cas system used to analyze naïve adaptation. The strain has deletions in *cas3, casC* and *cas1*, each indicated as Δ, resulting in loss of interference (‘Δ interference’) and loss of chromosomally encoded adaptation (‘Δ adaptation’). Adaptation was restored by expression of Cas1-Cas2 expression from pEB628 (lane 1, pCas1-Cas2), shown in an agarose gel of PCR reactions as expansion of CRISPR from 662 bp parental (P) to 723 bp expanded (E). Spacer acquisition was much reduced when D218A Cas1 replaced wild type enzyme (lane 2, pCas1^ND^-Cas2 for ‘nuclease defective’) and empty plasmid vector (pEmpty) showed no spacer acquisition (lane 3). Asterisks illustrate annealing positions of primers used in PCR reactions to detect CRISPR expansion (**B**) Summary agarose gel of CRISPR DNA from PCR reactions against genomic DNA from naïve cells that can acquire spacer when deleted for *recG, recA* or *ruv* (lanes 3 and 4 and 7–10), or cannot acquire spacer when deleted for *polA* and *recB* (lanes 5–6 and 11–12). (**C**) Agarose gels summarizing effects on primed adaptation in the *recG* deletion strain when expressing mutant RecG proteins from a plasmid (lanes 1–5), or RNaseHI inducibly expressed from the chromosome (Lanes 6 and 7). (**D**) Effects on primed adaptation of expressing helicase defective PriA (‘PriA300’) in Δ*recG* Δ*priA* cells (lanes 1 and 2), or of deleting primosome assembly proteins PriB, PriC or Rep (lanes 3–7).

Naïve adaptation assays (Figure 2A) followed a procedure similar to that in (5). Cells lacking Cas3, CasC and/or Cas1 and a priming spacer (spT3) were transformed by pEB628 expressing Cas1 and Cas2 or the empty plasmid as a control. Expression of Cas1-Cas2 was induced by addition of 0.2% (w/v) arabinose, and cells were sub-cultured three or four times by 1:300 dilution of the previous culture. Antibiotics were not included in these rounds of growth in LB to allow plasmid curing from spacer acquisition.

### Purification of *E. coli* proteins

Coomassie stained gels of purified proteins are shown in Supplementary Figure S3. Cas1 and Cas2 proteins were over-produced with N-terminal (His)_6_ tags in strain BL21 AI. Cells were grown at 37°C to optical density of 0.5–0.6 in LB ampicillin (50 μg/ml) and induced using arabinose (0.2% w/v), with growth continued for 3 h after induction. Cas1 or Cas2 expressing cells were harvested for re-suspension in buffer H (20 mM Tris-HCl pH7.5, 500 mM NaCl, 5 mM imidazole, 10% glycerol) for storage at −80°C prior to protein purification. The first purification step was identical for both Cas1 and Cas2: sonicated and clarified soluble cell extract was passed into a 5 ml Hi-Trap Nickel chelating column, Cas1 or Cas2 eluting within a gradient of increasing imidazole. Salt was reduced by dialysis into buffer H2 (20 mM Tris-HCl pH7.5, 100 mM NaCl, 10% glycerol, 1 mM DTT). For Cas1, fractions were loaded into a 5 ml Hi-Trap heparin column and eluted in a gradient of NaCl at 200–300 mM. Cas1 fractions were pooled for storage at −80°C in buffer H2 containing 40% glycerol. Cas1 mutant proteins D218A, R84G, R95G, R123G and R138G were purified in the same way as wild-type Cas1. Further rationale and details about these Cas1 arginine mutants are given in Supplementary Text and Figure S5. After the Nickel chelation step, Cas2 was loaded onto a S300 size exclusion column in buffer H2. Cas2 fractions were loaded onto a 5 ml heparin column and collected in the non-binding flow through or wash. Cas2 fractions were stored as for Cas1. RecG and PriA proteins were a gift from Prof. Bob Lloyd FRS (University of Nottingham) and RusA was purified (42).

### Assays on DNA

Base sequences of DNA strands used to construct substrates are given in the Supplementary Figure S5. DNA strands were custom synthesized and HPLC purified by Sigma-Aldrich. DNA strands (300 ng) were ^32^P labeled at their 5′ ends by incubation with T4 polynucleotide kinase (PNK) and γ ^32^P-ATP (1 h, 37°C) followed by heat inactivation of PNK. Unincorporated ATP was removed from these reactions using Bio-Spin 6 columns (Bio-Rad). Resulting end-labeled DNA was annealed to unlabeled DNA strands (900 ng) in buffer SSC (150 mM sodium chloride, and 15 mM sodium citrate, pH 7.0) by heating to 95°C for 2 min followed by gradual cooling to room temperature. DNA substrates were then purified, to remove un-annealed oligonucleotide or incomplete DNA structures, by electrophoresis through a 10% acrylamide Tris-Borate-EDTA (TBE) gel followed by autoradiography, excision of gel slice and elution by diffusion at 4°C into 250 μl of 10 mM Tris-HCl, 50 mM NaCl pH 7.5. Cas1/Cas2 binding to substrates was analyzed in electrophoretic mobility shift assays (EMSAs) through 5% acrylamide TBE gels at room temperature. Prior to electrophoresis, protein and DNA substrate were mixed for 10 min at ambient temperature in buffer SBHB (7 mM Tris-HCl pH 8.5, 9% glycerol, 50 mM NaCl, 100 μg/ml BSA) supplemented with 5 mM EDTA, in 20 μl reaction volumes. Gels were dried and exposed by phosphorimaging to detect ^32^P labeled DNA. Nuclease and end joining assays were in buffer SBHB supplemented with 10 mM magnesium chloride at 37°C for 10 min. Reactions were stopped by addition of 1 mg/ml proteinase K, 2.5% w/v SDS, formamide gel loading dye and heating to 75°C prior to electrophoresis through 15 or 20% acrylamide gels containing 5 M urea in 1xTBE buffer. Holliday junction and fork substrates *Chi* and *Chi^Sma^* were generated according to the method (43).

## RESULTS

### Differential requirements for RecG, PriA, RecB and DNA polymerase I in primed and naïve adaptation

We investigated adaptation in *E. coli* strains deleted for genes encoding proteins that help to maintain genome stability by DNA repair and homologous recombination. To assay primed adaptation we generated an *E. coli* strain derived from strains in references (32,41), described in Supplementary Table S1. This contained chromosomally inducible genes encoding Cas1, Cas2, Cas3 and Cascade proteins, and an inducible CRISPR spacer (spT3) (Figure 1A). Spacer spT3 encodes crRNA that has a perfect sequence match with the essential gene R in virulent lambda phage (λ*vir*) (41), but with a non-consensus PAM (5′-CCA), giving only partial protection against phage (44). Primed adaptation was detected as expansion of CRISPR after PCR amplification of genomic DNA extracted from cells surviving after infection with λ*vir*. Induction of chromosomal CRISPR-Cas resulted in expanded CRISPR consistent with addition of a single spacer-repeat unit (723 bp increased to 784 bp, Figure 1B lane 1). PCR and DNA sequencing of individual colony survivors with expanded CRISPR confirmed that new spacer sequences were acquired from λ*vir* (Supplementary Table S3).

Most gene deletions tested in primed adaptation assays had no observable effect on CRISPR expansion listed in Supplementary Table S4. Elimination of genes encoding RecG or PriA helicases (Δ*recG* or Δ*priA*) corresponded to loss of detectable CRISPR expansion (Figure 1B). DNA bands present in this, and subsequent, agarose gels were the only bands visible; untrimmed gels are shown in Supplementary Figure S1. We did not detect CRISPR expansion from DNA extracted from these deletion strains after infection with λ*vir*. Overall, λ*vir* infectivity was not significantly reduced by Δ*recG*, Δ*priA* or Δ*polA* (Supplementary Table S5A), consistent with the gene products acting on host cell adaptation rather than other events during phage infection. Loss of CRISPR expansion from Δ*recG* cells was restored by plasmid expression of RecG, but helicase inactive RecG Q640R (45) did not restore it (Figure 1B lanes 3–6). RecG helicase promotes genome stability in most species of bacteria (46,47), by rescuing stalled replication forks (43,48) and dissociating R-loops (49,50). Loss of primed adaptation from Δ*priA* cells was reversed when PriA or helicase inactive PriA (K230R, also called ‘*priA300*’) was expressed from a plasmid (Figure 1B lanes 10–12), giving at least two CRISPR expansion products, observations returned to later.

The DNA gap-filling enzyme DNA polymerase I, encoded by *polA*, was also essential for primed adaptation (Figure 1C). CRISPR expansion could not be detected when DNA synthesis activity of DNA polymerase I was lacking (Δ*polA*, Figure 1C lane 2). Expression of DNA polymerase I from a plasmid (pPolA^+^) restored spacer acquisition but empty plasmid vector did not (Figure 1C, lanes 3 and 4). Therefore, gene deletions in *recG, priA* or *polA* corresponded to a loss of primed adaptation, in contrast to deletions in other DNA recombination-repair genes that were proficient at primed adaptation (exemplified by Δ*recB* and Δ*ruvC*, Figure 1C lanes 5 and 6).

To test naïve adaptation interference was eliminated by deleting genes encoding Cas1, Cas3 and the major Cascade component CasC, and spacer spT3 was absent (Figure 2A and Supplementary Table S1A). Expansion of CRISPR by incorporation of a new spacer-repeat unit in naïve cells was detectable by inducible expression of Cas1-Cas2 from a plasmid (662 bp increased to 723 bp, Figure 2A lane 1), but no expansion was present after expressing catalytically defective Cas1 D218A (51), or empty plasmid (Figure 2A lanes 2 and 3). In contrast to primed adaptation, Δ*recG* cells did give detectable naïve adaptation, showing that RecG is dispensable for adaptation when interference is absent (summarized in Figure 2B with additional gels in Supplementary Figure S2). However, Δ*polA* naïve cells showed no detectable CRISPR expansion, indicating that it was required in both types of adaptation (Figure 2B and Supplementary Figure S2). We have been unable to test naïve adaptation in Δ*priA* cells because PriA is required for propagation of the Cas1-Cas2 plasmid. A recent report demonstrated crucial roles for RecBCD in supporting naïve adaptation when replication forks are collapsed (39). In our naïve adaptation assays, cells lacking RecB (Δ*recB*) also lacked detectable CRISPR expansion (Figure 2B lanes 5 and 6). RecBCD can initiate homologous recombination at DNA ends by providing a substrate for RecA to generate D-loops (52), which can be converted into Holliday junctions by RuvABC. However, *recA* and *ruvC* deletions did not abolish naïve or primed adaptation (Figure 2B and Supplementary Table S4). Therefore, these genetic data on *recB, recA* and *ruvC* indicate that naïve adaptation occurs independently of DNA double strand break repair, but is in agreement with RecBCD being required for DNA capture at collapsed forks when Cas3 nuclease is absent. This is also consistent with RecG being required for primed, but not naïve, adaptation because RecG acts independently of RecBCD recombination (42). We conclude that RecG helicase activity is required for primed adaptation in *E. coli*, which also requires the presence of PriA. DNA polymerase I is required for both primed and naïve adaptation. We examined in more detail possible roles of RecG and PriA in primed adaptation linked to blocked DNA replication forks that have not collapsed and therefore do not depend on homologous recombination.

### RecG and PriA acting at blocked replication forks enable primed adaptation

RecG and PriA control re-activation of blocked replication forks in bacteria (46,53). RecG and PriA mutants that give phenotypes associated with abnormal replication fork processing were tested for their ability to restore primed adaptation to Δ*recG* and Δ*priA* cells, when expressed from a plasmid. RecG mutated in a motif required for its physical localization to replication forks (R682A W683S; ‘RecG RW’ (54)), failed to restore CRISPR expansion (Figure 2C lanes 1–3). RecG RW is a fully functional helicase and fully complements UV or mitomycin C sensitivity of Δ*recG* cells (54). In contrast to RecG RW, a RecG mutation (ΔA693) that does form replication fork foci, but has reduced helicase activity and shows defective DNA repair *in vivo* (54), did restore CRISPR expansion (Figure 2C lane 4). For comparison, also shown is helicase inactive RecG Q640R that did not support primed adaptation (lane 5, Figure 1). These phenotypes are consistent with RecG promoting primed adaptation at replication forks in a way distinct from DNA repair. RecG also dissociates R-loops (50,55), therefore, we examined if this activity is important for primed adaptation. To do this we utilized R-loop degradation by RNaseHI, which has the same effect overall as R-loop dissociation by RecG. Significantly, primed adaptation that had been abolished in *E. coli* Δ*recG* cells was restored when RNaseHI was inducibly over-expressed from an engineered chromosomal cassette (Figure 2C, lanes 6 and 7). These genetic data suggest that two known biological roles for RecG, association with replication forks and removal of R-loops, enable primed adaptation.

PriA re-activates arrested DNA replication by helicase-dependent and helicase-independent pathways that are antagonized by RecG to control against pathological over-replication (46,56,57). Helicase inactive PriA (‘PriA300’) supported primed adaptation in Δ*priA* cells (Figure 1D), so we assessed helicase independent roles of PriA in more detail. We first observed that the PriA300 allele restored primed adaptation in Δ*recG* Δ*priA* cells (Figure 2D lanes 1 and 2), consistent with a replication fork re-activation phenotype of PriA300 in Δ*recG* Δ*priA* cells (56). Multiple CRISPR expansion products were not observed in these cells, in contrast to cells lacking only *priA* shown in Figure 1D (lane 5), discussed later. PriA300 can orchestrate replication restart without helicase activity by re-loading DnaB replicative helicase *via* interactions with other proteins, PriB or PriC and Rep (56,58). Individual deletions of *priB*, *priC* or *rep*, had no observable effect on primed adaptation (Figure 2D, lanes 3–7). Primed adaptation could also be observed when double deletion of *recG* and *priA* was combined with *priC* (i.e. Δ*recG* Δ*priA* Δ*priC*) in cells expressing pPriA300 (Supplementary Figure S2B). We were unable to construct strains with combinations Δ*recG* Δ*priA* Δ*priB* or Δ*recG* Δ*priA* Δ*rep* even with the presence of pPriA300, which may relate to the previously noted inviability of these combinations of chromosomal deletions (59,60). These data indicate a role for PriA in primed adaptation that is not as a helicase or replisome re-loader.

### Binding of Cas1 and Cas1-Cas2 to forked DNA containing single stranded gaps

**Figure 3.**
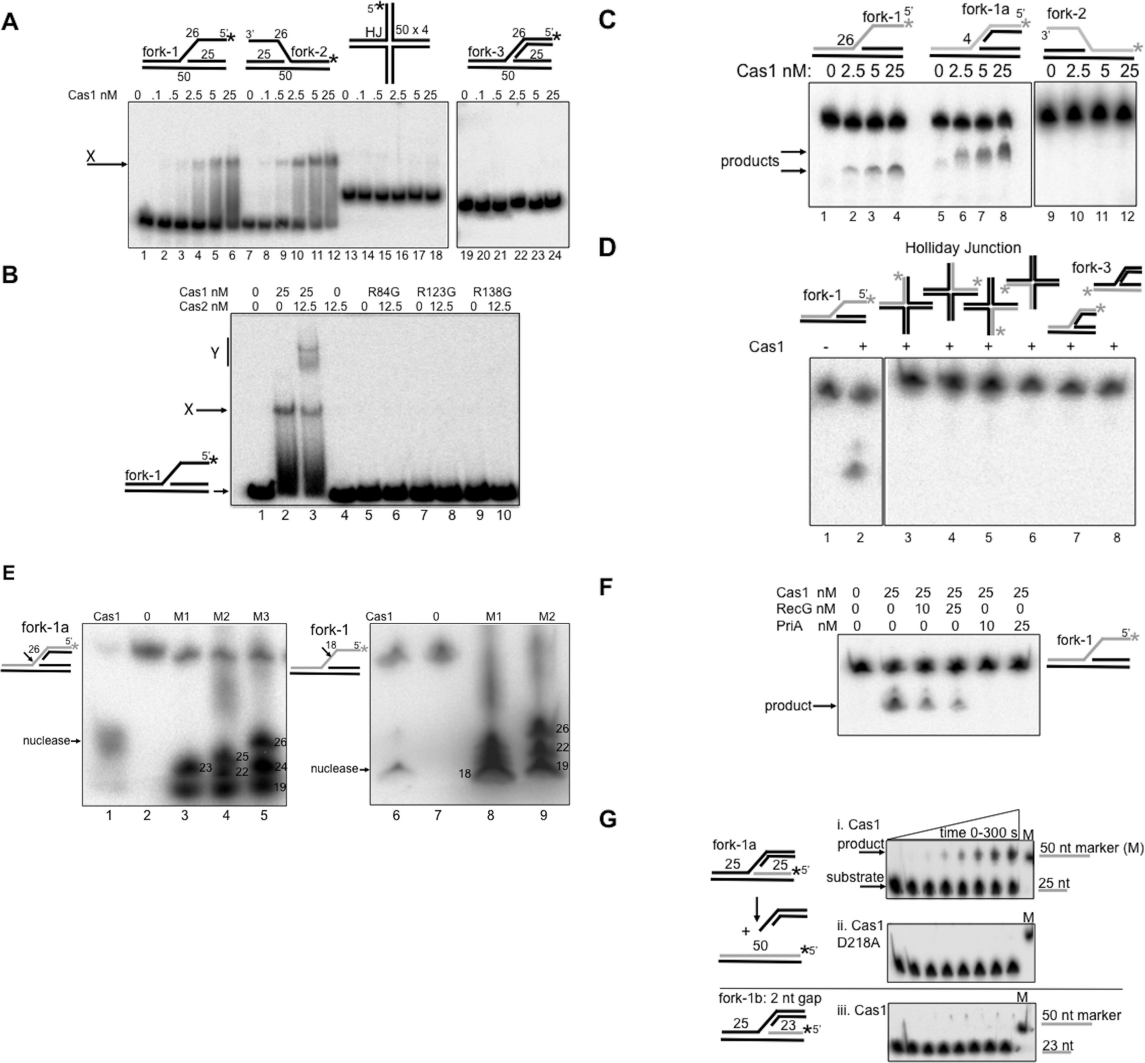
DNA binding and catalysis by *E. coli* Cas1 and Cas2. In each panel (A–F) an asterisk (*) denotes the position of the ^32^P label at 5′ DNA ends of appropriate DNA strands. (**A**) EMSAs of Cas1 binding to branched DNA substrates. Cas1 monomer protein concentrations are given above the panels, in reactions containing 6 nM of DNA. ‘X’ indicates stable Cas1-DNA complex observed with forks. Nucleotide (nt) lengths of DNA strands in fork substrates are indicted (25 or 50 nt), and 26 nt for the ssDNA flap regions of Fork-1 or Fork-2. (**B**) EMSA showing the effect of pre-mixing Cas1 with Cas2 on binding to fork-1, at protein monomer concentrations indicated above the panel, in reactions containing 6 nM of DNA. ‘X’ denotes defined Cas1-DNA complex, and ‘Y’ a second complex requiring Cas1 fork binding in the presence of Cas2. Lanes 5–10 show that complex Y is not formed when Cas1 mutant proteins (R84G, R123G or R138G) were pre-incubated with Cas2 as indicated because each is unable to bind to the fork DNA. (**C**) Urea denaturing gels showing products from nicking of fork DNA (6 nM) by Cas1 (2.5–25 nM). Numbers 26 and 4 refer to the nucleotide length of the ssDNA region on the labeled DNA strand of each fork. (**D**) Urea denaturing gel comparing product of Cas1 (25 nM) nicking fork-1 (lane 2) to lack of detectable product from any strand of Holliday junction (lanes 3–6) or fully base paired fork (lanes 7 and 8). (**E**) Urea denaturing gels showing product of Cas1 (25 nM) nicking fork-1a (6 nM, lanes 1 and 2) or fork-1 (lanes 6 and 7) compared to ^32^P end labeled marker DNA strands (M1–M3) of known nucleotide length, indicated on the gel panel. Arrows and numbers next to each fork substrate show the position of nicking. (**F**) Urea denaturing gel showing that pre-mixing of Cas1 with PriA, as indicated above the panel, results in loss of detectable Cas1 nicking fork-1. (**G**) Urea gels summarizing strand joining by Cas1 (25 nM) on forked DNA (6 nM) labeled on the fork leading strand (panel i), generating a 50 nt product from joining the 25 nt lagging strand of the fork with the 25 nt leading strand. Activity is lost when Cas1 is catalytically inactive (panel ii), or if the leading strand 3′ OH group is recessed two nucleotides away from the fork branch point (panel iii).

Purified *E. coli* Cas1 and Cas2 proteins (Supplementary Figure S3) were tested for binding to DNA fork and Holliday junction substrates that mimic structures generated at blocked replication forks. Illustration of the forks used and their full nucleotide composition is given in Supplementary Figure S4. We used Cas1 protein at 0.1–25 nM (monomer) concentration, at least 10-fold lower concentration than reported previously (51). In EMSAs Cas1 bound as a stable complex to forks containing 25 nucleotides (nt) of single-stranded DNA (ssDNA) (respectively, ‘fork-1’/’fork-2′) (Figure 3A, lanes 1–12), but binding was barely detectable using an equivalent fully base-paired fork (‘fork-3’) or a Holliday junction (lanes 13–24). Pre-incubation of Cas1 with Cas2 prior to mixing with fork-1 DNA gave a super-shifted EMSA complex (Figure 3B, lane 3, labeled Y) but Cas2 alone was unable to bind fork-1 (lane 4). We also purified three mutant *E. coli* Cas1 proteins (R84G, R123G and R138G) that we newly identified as being defective in spacer acquisition when expressed instead of wild-type Cas1 in *E. coli*, detailed in Supplementary Results and Supplementary Figure S5. Each mutant protein was unable to bind fork-1 and did not form complex Y when Cas2 was added (Figure 3B, lanes 5–10). Therefore, complex Y seems to represent stable binding of Cas1 with Cas2 to the fork DNA, consistent with requirement for a Cas1-Cas2 complex in CRISPR adaptation (36). No Cas1-Cas2 complex was observed in EMSAs using Holliday junction or fully based-paired fork-3, substrates that were also not bound by Cas1 alone (data not shown).

### Cas1 nicks forked DNA within single stranded gaps

Catalytic activities of Cas1 (37,51) were also investigated on DNA forks and Holliday junctions using 0.1–25 nM of protein and the addition of magnesium to reactions. We assessed in denaturing gels whether Cas1 could nick individual DNA strands in these substrates that would indicate the potential to collapse a fork or resolve a Holliday junction. Cas1 nicking was observed on the fork-1 strand containing 25 nt ssDNA, and on the same strand of a derivative of fork-1 (fork-1a) containing only 4 nt of ssDNA at the branch point (Figure 3C). Catalytically inactive Cas1 D218A gave no assay product (Supplementary Figure S6A). Cas1 was inactive on fork-2 even though its ssDNA nucleotide sequence was identical to fork-1 ssDNA though of opposite polarity (Figure 3C, lanes 9–12). There was no detectable nicking activity on the identical DNA strand within Holliday junction or fully base-paired fork (fork-3) (Figure 3D lanes 3–8) and Cas1 activity on the corresponding ssDNA alone or within a flayed duplex was barely detectable compared to Fork-1 (Supplementary Figure S6B). The inability of Cas1 to cut fork-3 and Holliday junction in these assays was confirmed in alternate assays using *Chi* and *Chi*^Sma^, large substrates sensitive for detecting structure-specific resolution of Holliday junctions and forks by nucleases (43), when compared to a *bona fide* resolving enzyme RusA (61) (Supplementary Figure S6C).

Cas1 nicked fork-1 and fork-1a within single stranded DNA (ssDNA) at 18 and 26 nucleotides from the ^32^P-labeled 5′-DNA end (Figure 3E). Nicking of fork-1a was within the ssDNA gap at the branch point, showing that it does not require availability of a ssDNA end for nicking activity. Binding of Cas1 to fork-1a in EMSAs was similar to binding of fork-1 (Supplementary Figure S6D). Since pre-incubation of *E. coli* Cas1 with Cas2 formed complex Y (Figure 3B) we also tested if Cas2 stimulated the Cas1 nicking activity on fork-1, which was maximally 20% product from 25 nM Cas1. However, Cas2 had no effect on nicking by Cas1 on any substrate, and Cas2 alone had no detectable nuclease activity (Supplementary Figure S5E). Interestingly, pre-mixing of Cas1 with PriA abolished Cas1 nicking of fork-1, but Cas1 was still active if RecG was added instead of PriA (Figure 3F). We conclude that Cas1 assayed at low concentrations can bind and nick fork DNA in single strand DNA gaps (e.g. fork-1a), with high specificity compared to other branched DNA. This activity of Cas1 could collapse a fork, generating DNA ends for processing and capture during CRISPR adaptation.

**Figure 4.**
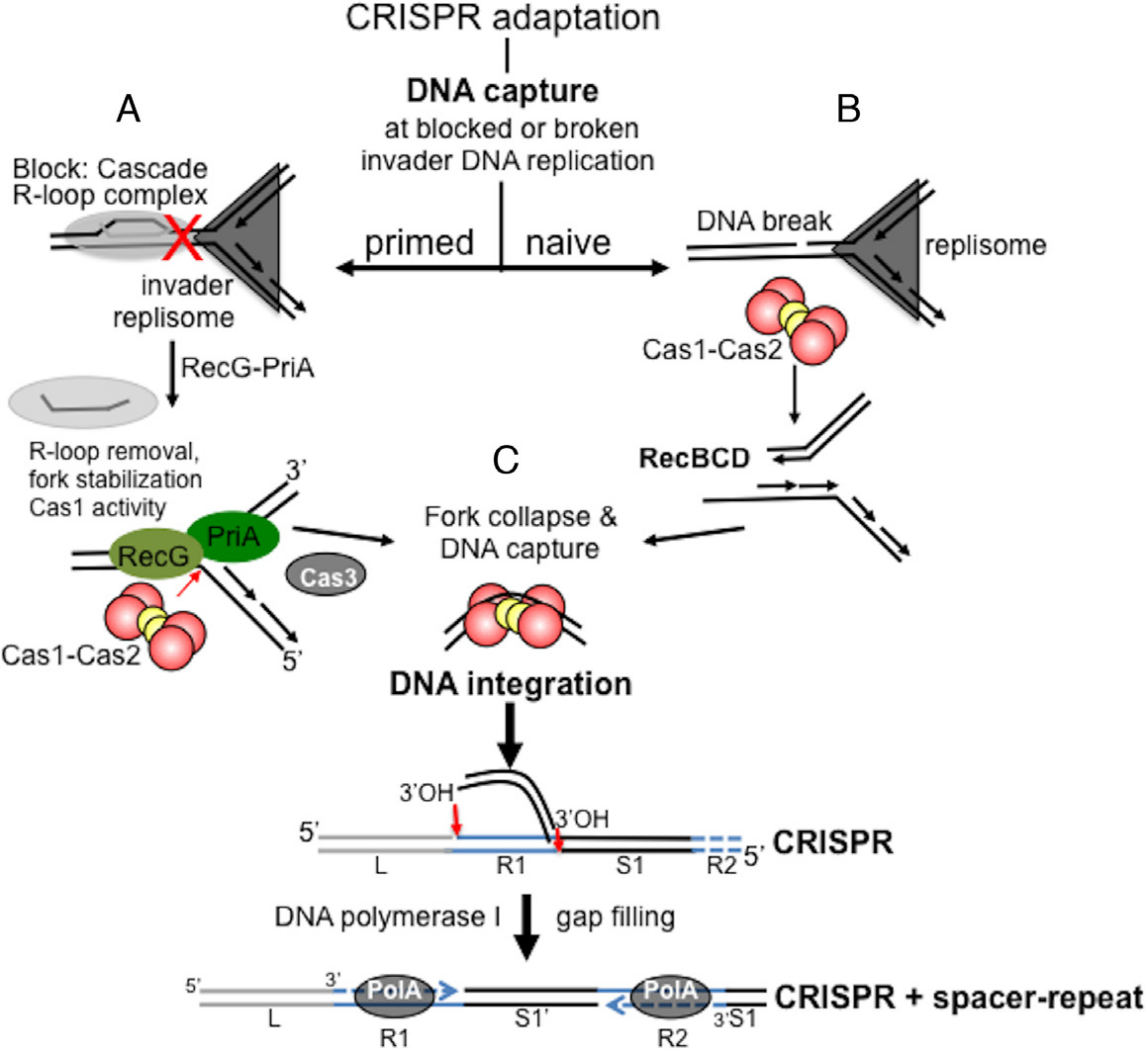
Model proposing different requirements for DNA capture in primed and naïve adaptation *E. coli*. Cas1-Cas2 is depicted according to the atomic resolution crystal structure PDB accession 4P6I and reference 74. DNA capture is suggested to require invader DNA replication forks that are compromised according to the presence or absence of Cascade. (**A**) In primed adaptation Cascade R-loop complexes block advancing invader DNA replication forks as indicated by the symbol X. RecG and PriA identify such blockages. PriA binding to the fork 3′ end limits fork-remodeling activities, discussed in the text, until removed by RecG helicase activity that remodels forks and removes R-loops. Cas1 is presented with an invader fork substrate for nicking and DNA capture. This could collapse the invader DNA replication fork. Further nucleolytic processing of DNA, possibly by Cas1 cutting a fork more than once, or by actions of Cas3 may be required to liberate DNA for the capture step. (**B**) In naïve adaptation, forks that are collapsed by Cas1 nicking, or by lesions or collisions, are processed by RecBCD (39). This generates invader DNA for capture. (**C**) DNA polymerase I, indicated as ‘PolA’ was required for both naïve and primed adaptation. Its polymerase activity can fill single strand DNA gaps (62), an activity that may aid DNA capture by generating duplex DNA after processing of invader DNA into ssDNA regions. Alternatively, or additionally, DNA polymerase I could act during new spacer integration (S1’) described. Integration leaves DNA repeat gaps (R1 and R2) flanking the new spacer (S1’), requiring synthesis of new DNA yielding one new repeat (R1). DNA polymerase I may catalyze this synthesis similar to its ‘gap filling’ role in DNA repair. The cruciform DNA structure that Cas1-Cas2 is thought to target in CRISPR for spacer integration is omitted for clarity.

End labeling of fork-1a on alternative strands revealed Cas1 catalyzed strand joining of the leading strand 3′ OH DNA to the 5′ end generated from Cas1 nicking in ssDNA of the same fork (Figure 3G). Strand joining in the fork was lost if Cas1 D218A was used, or if the leading strand 3′ OH group was located two nucleotides (or more) away from the fork branch point (fork-1b, Figure 3H panels (ii) and (iii)). Cas1 catalyzed transesterification reactions on fork substrates were recently detailed in (68), and are required for spacer integration into CRISPR, exemplified by integration of a radiolabeled duplex DNA fragment into a supercoiled plasmid (37), a reaction also supported by Cas1 in this study (Supplementary Figure S7). Cas1 strand joining reactions are unlikely to occur at blocked replication forks because the necessary 3′ OH would be located >2 nucleotides away from the cut branch point. Instead, fork nicking by Cas1 may enable DNA capture at replication forks, for strand joining during spacer integration at CRISPR loci, each event aided by the identified host factors as discussed below and in Figure 4.

## DISCUSSION

We present new insights into how genome stability systems underpin the building of CRISPR-Cas immunity by either primed or naïve adaptation in *E. coli*. In primed adaptation, Cascade-Cas3 interference complexes that are not proficient in degrading invader DNA stimulate adaptation as a means to update immunity by acquisition of new spacers (32). In naïve adaptation immunity can be established by Cas1-Cas2 without interference reactions (5). A striking outcome of our genetic analysis was that primed adaptation required RecG as an active helicase that can localize to replication forks, but naïve adaptation did not. Conversely, RecB, that is integral to RecBCD complex, was required for naïve adaptation but not primed. Further insight into adaptation was given by identification that DNA polymerase I was required for both primed and naïve adaptation. Our interpretation of these data is that DNA polymerase I is required for DNA synthesis common to both naïve and primed adaptation, but that requirements for RecBCD and RecG-PriA reflect their interactions with different DNA substrates that may arise at replication fork damage associated with actions of Cas proteins targeted to invader DNA. A recent report on naïve adaptation identified that modulation of RecBCD nuclease–translocase activity in response to *Chi* DNA sequence is critical for specifying invader DNA for capture (39). In primed adaptation Cascade interference complexes would act as a mechanism specifying invader DNA, acting as ‘programmed’ roadblocks to invader replication, triggering RecG helicase activity to remove the blockages, exposing DNA for capture.

A model for involvement of genome stability proteins in underpinning adaptation is presented in Figure 4, summarized into three parts. (i) In primed adaptation, Cascade R-loop complexes block invader DNA replication. RecG and PriA respond, with RecG helicase activity dissociating the R-loop by unwinding RNA–DNA hybrids and removing bound proteins, including possibly Cascade, PriA and SSB. This remodels the blocked replication fork into exposed double and ssDNA regions for DNA capture by the catalytic activity of Cas1. Cas3, which is also essential for primed adaptation (32), may also contribute to generating DNA fragments at this stage. (ii) In naïve adaptation RecBCD nuclease–helicase activity resects DNA ends generated by collapsed invader replication forks, independently of Cascade. This generates DNA substrate for capture. Fork nicking by Cas1 (Figure 3) could be responsible for collapse of forks. (iii) DNA polymerase I catalyzes ‘gap filling’ DNA synthesis (62) of a new CRISPR repeat during spacer integration.

Cascade R-loops have potential to act as replication roadblocks similarly to stalled RNA polymerase and other protein–DNA complexes (63–65). It was significant that primed adaptation required RecG helicase activity and a functioning RecG fork localization motif. Additionally, primed adaptation defects caused by elimination of RecG could be corrected by ectopic over-expression of RNaseHI (Figures 1 and 2). Therefore, removal of replication fork-blocking R-loop nucleoprotein complexes by RecG helicase is implicated as being crucial for the mechanism of primed adaptation, in line with other reported roles for RecG (50,54,55). Future experiments to ascertain directly if Cascade can block DNA replication will require *in vitro* reconstitution of the replisome based on previous studies in *E. coli* (63,66). Another activity of RecG, conversion of forked DNA into a Holliday junction structure (43,67), was initially appealing to us for facilitating primed adaptation, by Cas1 cleaving a Holliday junction to generate ends for DNA capture. However, Cas1 in our assays (0–25 nM Cas1: 6 nM of DNA) showed strong preference for binding to and nicking fork substrates with ssDNA gaps. Cas1 was inactive on large *Chi* structures that represent Holliday junction or fully base-paired fork DNA, giving further evidence that Cas1 prefers branched DNA substrates that are at least partially ssDNA. Cas1 assayed at much greater monomer concentrations (250 nM to low μM) was observed to cleave similar structures most efficiently in a previous analysis (51), although we did not observe any nicking of Holliday junction or fully base-paired fork DNA. The nicking activity of Cas1 that we observed on ssDNA forks is in line with a very recent analysis of a Cas1-Cas2-forked DNA complex presented at atomic resolution (74).

Binding of Cas1, and Cas1-Cas2, to forks containing ssDNA gave distinct stable in-gel complexes. Binding was lost when using any of the acquisition defective Cas1 mutants R84G, R123G or R138G (Figure 3B and Supplementary S5). Cas1 collapsed the same forks by nicking DNA within the single strand gap. Cas1 catalyzes transesterification, or dis-integration, reactions that join together DNA strands (37). Strand joining by Cas1 was also efficient within forked DNA, shown here and in (68). DNA strand joining by Cas1 is crucial for incorporation of new spacer DNA into a CRISPR locus, and utilizes DNA sequence specificity that matches integration sites for new spacers at the leader end of an *E. coli* CRISPR (68). Blocked replication forks are unlikely substrates for Cas1 strand joining reactions because they lack DNA 3′ OH located exactly proximal to the fork branch point. Therefore, in that context strand joining would not be possible in the way it can take place during spacer integration into CRISPR. Therefore, Cas1 may be versatile during adaptation, by nicking and collapsing forks for DNA capture, and joining DNA strands when suitable ends are available for transesterification in CRISPR. In conclusion we suggest that requirement for RecG helicase in primed adaptation centers on aiding Cas1 in capture of protospacer DNA, rather than DNA integration into a CRISPR locus, because if RecG DNA helicase were required for integration this would be expected to correlate with impaired naïve adaptation in Δ*recG* cells, which also need integration to occur, but this was not the case.

PriA fully inhibited Cas1 activity, a surprising observation given that PriA was required for primed adaptation. PriA binds to fork branch points in a position to accommodate the leading strand 3′ end into a binding pocket (69,70), which would most likely block access to the fork branch-point by Cas1. However, PriA binding may also limit fork conversion into substrates for homologous recombination (57,71), an effect that could be advantageous for primed adaptation if Cas1 nicks an intact fork. We also observed that plasmid expression of PriA or PriA300 in RecG^+^ primed adaptation cells gave additional CRISPR expansion products (Figure 1D), but did not when RecG was absent (Figure 2D). We speculate that this may reflect increased mobilization of RecG helicase to blocked forks in response to artificially increased PriA levels, with the corresponding enhancement of adaptation. Antagonism between RecG and PriA is important for maintaining genome stability in bacteria when replication forks stall or require termination at *ter* sites (46,72,73). They may be a factor in the observed bias toward spacer acquisition from *ter* sites observed in a recent study, although that was not tested (39). RecG and PriA are present in most species of bacteria, including those utilizing Cas9 for interference, raising the possibility that primed adaptation enabled by these helicases responding to blocked replication may be widely relevant.

## Supplementary Material

SUPPLEMENTARY DATA
