# Supplementary material for: Different genome stability proteins underpin primed and naïve adaptation in E. coli CRISPR-Cas immunity
Source: Nucleic Acids Res. 2015 Nov 17;43(22):10821–30. doi: 10.1093/nar/gkv1213 (PMC4678826; doi:10.1093/nar/gkv1213)
Supplement: SUPPLEMENTARY DATA [file supp_43_22_10821__index.html]

Different genome stability proteins underpin primed and naïve adaptation in E. coli CRISPR-Cas immunity — SUPPLEMENTARY DATA 

# Different genome stability proteins underpin primed and naïve adaptation in *E. coli* CRISPR-Cas immunity

## SUPPLEMENTARY DATA

- SUPPLEMENTARY DATA
