# Supplementary material for: Different genome stability proteins underpin primed and naïve adaptation in E. coli CRISPR-Cas immunity
Source: Nucleic Acids Res. 2015 Nov 17;43(22):10821–30. doi: 10.1093/nar/gkv1213 (PMC4678826; doi:10.1093/nar/gkv1213)
Supplement: SUPPLEMENTARY DATA [file supp_gkv1213_nar-01977-h-2015-File010.pdf]

SUPPLEMENTARY RESULTS AND METHODS

Recombineering and Transductions and strain manipulations for genetic analysis of spacer acquisition

Gene deletions were made using “recombineering” [1], generating a PCR cassette to target the desired gene for insertional inactivation by an antibiotic resistance gene and plasmid expression of a recombinase (pKD46) followed by purging the plasmid by growing cells at 37°C. Transductions were used to move around antibiotic resistance gene deletion cassettes using standard methods from P1 lysates, briefly: An overnight culture of the strain to be transduced was inoculated into 8 mL fresh broth and grown to optical density of 0.8. Pelleted cells were resuspended for 10 minutes at ambient temperature in 1 mL buffer MC (100 mM $MgSO_4$, 5 mM $CaCl_2$), prior to addition of P1 lysate of various titers prepared to contain the desired selectable gene cassette. Incubation, at typically 37°C for 30 minutes, was followed by addition of sodium citrate to 1 mM, followed by suspension of the P1-*E. coli* mixture in warm liquid 0.6% agar broth and plating onto agar containing the appropriate antibiotic selection. Incubation was at 37°C for up to 48 hours to allow growth of resistant colonies that were then purified by antibiotic selection and verified for correct insertion of the desired gene cassette.

TABLE S1A: *E. coli* strains reported in the main results of this work. In summary,
IIB969 was used for primed adaptation, made by transduction of cassette *cas3-cascade-cas1-cas2-CRISPR* from BW40114 into BW39651 [2] [3]. IIB970 and EB366 were used for naïve adaptation, made from BW25113 [1] by transducing in *araB*::T7RNAp (T7 RNA polymerase) to generate IIB924 that was further modified by deletion of *cas3* and *casC*, encoding the interference nuclease-helicase Cas3 and the major structural subunit of Cascade CasC. Additionally, in EB366, *cas1* was deleted.

Bacterial strain	Relevant genotype	Source and reference
BL21-AI	F^- *ompT hsdS*$_B$(r_B-m_B-*) gal dcm araB*::*T7RNAP-tetA*	Invitrogen^{TM}
EB304	MG1655 Δ*cas3*::*apra*	[4]

N3072	+ Δ*recA*::*Cm*	Gift from R. G. Lloyd
N4634	+ Δ*recB270*::*kan*	Gift from R. G. Lloyd
JC19009	+ *priA2*::*kan*	[5]
AM2017	+ Δ*lacIZYA* Δ*priB*::*dhfr*	[6]
N6052	+ Δ*recG1919*::*apra*	*E. coli* stock center
BW25113	Δ(*araD-araB*)*567* Δ*lacZ4787*(::*rrnB-3*) λ$^-$ *rph*-1, Δ(*rhaD-rhaB*)*568 hsdR514*	[1]
	<u>*Strains derived from BW25113*</u>	
BW39651	+ λT3	[2]
BW40114	+ F' (*proAB*$^+$*lacI*^{q}ZΔM15::Tn*10*) *lac*UV5-*cas3 cat*::*araB*p8-*casA*	[3]
IIB848	+ λT3 Δ*cas3*::*apra*	recombineering with pKD46
IIB859	+ λT3 Δ*casC*::*kan*	recombineering with pKD46
JW2728-2	+ Δ*casC760*::*kan*	Keio collection
BW39183	+ Δ*cas1*::*kan*	Gift from E. Semenova
IIB868	+ Δ*cas3*::*apra*	P1. EB304 x BW25113
CGSC8612	+ Δ*priC752*::*kan*	*E. coli* Stock Center
CGSC11594	+ Δ*rep729*::*kan*	*E. coli* Stock Center
IIB888	+ Δ*cas3::apra* Δ*casC760::kan*	P1.JW2728-2 x IIB868
IIB892	+ Δ*cas3*::*apra* Δ*casC760*::*kan*^{S}	Removal of kan by pCP20
IIB924	+ *araB*::T7RNAp	P1. BL21-AI x BW25113
IIB969	+ λT3 *lac*UV5-*cas3 cat*::*araB*p8-*casA*	P1. BW40114 x BW39651
EB358	+ λT3 *lac*UV5-*cas3 cat*::*araB*p8-*casA* Δ*cas1*::*kan*	
IIB970	+ *araB*::T7RNAp Δ*cas3*::*apra* Δ*casC*::*kan*^{s}	P1. IIB892 x IIB924
IIB971	+ *araB*::T7RNAp Δ*cas3*::*apra* Δ*casC*::*kan*^{s} Δ*recA*::*Cm*	P1. N3072 x IIB970
IIB981	+ *araB*::T7RNAp Δ*cas3*::*apra* Δ*casC*::*kan*^{s} Δ*recB270*::*kan*	P1. N4634 x IIB970
IIB997	+ Δ*recG263*::*kan cat*::*araBp8-casA*	P1. N4256 [7]x BW25113

	+ Δ*recG263*::*kan* + λT3 *lac*UV5-*cas3*	
IIB998	*cat*::*araB*p8-*casA*	P1. IIB969 x IIB997
IIB1012	+ *polA*::EZ*kan* + λT3 *lac*UV5-*cas3* *cat*::*araB*p8-*casA*	P1. JJ1038 [8] x IIB969
IIB1020	+ Δ*priA2*::*kan* + λT3 *lac*UV5-*cas3* *cat*::*araB*p8-*casA*	P1. JC19009 [5] x IIB969
IIB1032	Δ*cas3*::*apra* + λT3 *lac*UV5-*cas3* *cat*::*araB*p8-*casA*	P1.IIB848 x IIB969
IIB1033	Δ*casC*::*apra* + λT3 *lac*UV5-*cas3* *cat*::*araB*p8-*casA*	P1. IIB859 x IIB969
EB366	+ Δ*cas1*::*kan* Δ*cas3*::*apra* Δ*casC*::*kan*^{s} *araB*::T7RNAp	P1. BW39183 x IIB970
IIB1044	*polA*::EZ*kan*^{S} Δ*cas3*::*apra* + Δ*casC*::*kan*^{s} Δ*cas1*::*kan* *araB*::T7RNAp	P1. JJ1038 [9] x EB366
IIB1045	+ Δ*recG263*::*kan*^{S} Δ*cas3*::*apra* + Δ*casC*::*kan* Δ*cas1*::*kan* *araB*::T7RNAp	P1. N4256 [7] x EB366
IIB1046	+ Δ*priA2*::*kan*^{S} Δ*cas3*::*apra* + Δ*casC*::*kan* Δ*cas1*::*kan* *araB*::T7RNAp	P1. JC0019 [5] x EB366
IIB1069	+ λT3 *lacUV5-cas3* Δ*priC752*::*kan* *cat*::*araBp8-casA*	P1.CGSC8612 x IIB969
IIB1071	+ λT3 *lacUV5-cas3* *cat*::*araBp8-casA* Δ*rep719*::kan	P1.CGSC11594 x IIB969
IIB1081	+ λT3 *lacUV5-cas3* *cat*::*araBp8-casA* Δ*priB*::*dhfr*	P1.AM2017 [6] x IIB969
IIB1085	+ λT3 *lac*UV5-*cas3* *cat*::*araB*p8-*casA* Δ*recG1919*::*apra*	P1. N6052 or CGSC12491 x IIB969
IIB1086	+ΔpriA2::kan + λT3 lacUV5-cas3 cat::araBp8-casAΔrecG1919::apra +pPriA300	P1. N6052 x IIB1020 containing pPriA300
IIB1089	+ λT3 *lac*UV5-*cas3* *cat*::*araB*p8-*casA* Δ*recG1919*::*apra* Δ*proB*::ParaBAD *rnhA*$^{+}$-frt > *kan* > frt	P1.RCe442 x IIB1085
RCe442	MG1655 Δ*proB::* ParaBAD *rnhA*$^{+}$-frt > *kan* > frt	[10]

IIB1093	+ λT3 *lac*UV5-*cas3 cat*::*araB*p8-*casA* Δ*recG1919*::*apra* Δ*priC*::*kan*	P1. CGSC8612 x IIB1085
IIB1096	+ λT3 *lac*UV5-*cas3 cat*^{s}::*araB*p8-*casA* Δ*recG1919*::*apra* Δ*priC*::*kan*^{s}	Removal of kan and chl cassette by pCP20
IIB1098	+ λT3 *lac*UV5-*cas3 cat*^{s}::*araB*p8-*casA* Δ*recG1919*::*apra* Δ*priC*::*kan*^{s} *priA2*::*kan* + pPriA300	P1. JC19009 x IIB1096 + pPriA300

TABLE S1B. Other strains used in this work, presented as results in Table S1.

Bacterial strain	Relevant genotype	Source or reference
IIB976	+ λT3 *lac*UV5-*cas3 cat*::*araB*p8-*casA* *recB268*::Tn*10*	P1.N3071 [11] x IIB969
IIB990	+ λT3 *lac*UV5-*cas3 cat*::*araB*p8-*casA* Δ*hptG*::*kan*	P1. JW0462 [12] (Keio) x IIB969
IIB992	+ λT3 *lac*UV5-*cas3 cat*::*araB*p8-*casA* *recJ2052*::Tn*10kan*	P1.LMM1032 [13] x IIB969
IIB993	+ λT3 *lac*UV5-*cas3 cat*::*araB*p8-*casA* *recF400*:Tn*5*	P1. WA576 [12] x IIB969
IIB994	+ λT3 *lac*UV5-*cas3 cat*::*araB*p8-*casA* Δ*hns*::*kan*	P1. BW39121 [2] x IIB969
IIB996	+ λT3 *lac*UV5-*cas3 cat*::*araB*p8-*casA* *recQ1803*::*Tn3*	P1. IRB101 [14] x IIB969
IIB1006	+ λT3 *lacUV5-cas3 cat::araBp8-casA* Δ*ruvABC*::apra	P1.AM1955 [8] x IIB969
IIB1008	+ Δ*rnhA*733::kan λT3 *lacUV5-cas3 cat::araBp8-casA*	P1. IIB969 X IIB776
IIB1013	+ λT3 *lacUV5-cas3 cat::araBp8-casA* Δ*recA*1921::spec	P1.AM1986 x IIB969
IIB1024	+ λT3 lacUV5-cas3 cat::araBp8-casA *sbcD*::km	P1.LMM1247 [15] x IIB969
IIB1031	+ λT3 lacUV5-cas3 cat::araBp8-casA Δ*recE*787::kan	P1.JW1344 [12] x IIB969

TABLE S2: Plasmid constructs used for genetic analysis and protein purification.

Plasmid	Description/Reference
pRecG^{+} (pEB615)	*E. coli* RecG^{+} in pUC19.
pRecG Q630R (pEB620)	Helicase inactive RecG in pUC19.
pRecG 'RW' R682A W683S (pEB641)	RecG defective in formation of replication fork foci [16], in pUC19.
pRecG 'ΔC1' ΔA693 (pEB640)	DNA repair defective RecG [16], in pUC19.
pPriA^{+} (pEB621)	*E.coli* PriA^{+} in pUC19.
pPriA300	Helicase inactive PriA K230R in pET-3c [17].
pPolA^{+} (pEB588)	*E. coli* DNA polymerase I in pUC18.
pCas1-Cas2 (pEB628)	*E. coli* Cas1 and Cas2 in pBad-HisA.
pCas1 D218A-Cas2 (pEB639)	Catalytically inactive Cas1 D218A with wild type Cas2 in pBad-HisA.
pCas1 R84G pSJW5	Cas1 R84G with wild type Cas2 in pBad-HisA.
pCas1 R95G pSJW6	Cas1 R95G with wild type Cas2 in pBad-HisA.
pCas1 R123G pSJW7	Cas1 R123G with wild type Cas2 in pBad-HisA.
pCas1 R138G pSJW8	Cas1 R138G with wild type Cas2 in pBad-HisA.
p$(His)_6$Cas1 (pEB505)	*E. coli* Cas1 in pET14b for purification of N-terminally hexahistidine tagged Cas1.
pCas1 D218A (pASB22)	*E. coli* Cas1 D218A in pET14b as for wild type. Encodes catalytically inactive Cas1 protein.
pCas1 R84G (pSJW9)	*E. coli* Cas1 R84G in pET14b.
pCas1 R95G (pSJW10)	*E. coli* Cas1 R95G in pET14b.
pCas1 R123G (pSJW11)	*E. coli* Cas1 R123A in pET14b.
pCas1 R138G (pSJW12)	*E. coli* Cas1 R138A in pET14b.
p$(His)_6$Cas2 (pASB3)	*E. coli* cloned into pET14b for purification of N-terminally hexahistidine tagged Cas2.
E. coli CRISPR-1 cloned into pBluescript (pJRW2)	PCR of *E. coli* MG1655 CRISPR-1 for cloning *via* BamHI and XbaI sites.

TABLE S3: Sequences of new spacers in CRISPR-1 identified from testing IIB969, alluded to in results.

**Acquired spacer sequence 5' to 3'**	**Protospacer match**	**strand**	**SAM**	**32-35bp**	**length**
CCAGAATGCAGAATCACTGGCTTTTTTGGTTG	*rexB*	**-**	**AAG**	**TGC**	**32**
CGGAGAGGATGAATGACGCGACAGGAAGAA	*A* and *W*	**+**	**ATC**	**CTT**	**30**
ATATTCTGGCAAAAAATTCCGTTGCAGATGTT	*orf-314*	**+**	**GGG**	**CTT**	**32**
AGCTTTTACTGCTTCGGCCTGTGTCAGTTCTG	*bet*	**-**	**AAG**	**ACG**	**32**
TGGACCCAACTCGAAATCAACCGTAACAAGCA	*cII*	**+**	**AAG**	**ACA**	**32**

TABLE S4: Gene deletions in *E. coli* that did not abolish either primed or naïve adaptation during infection of with phage λvir. In each gene deletion strain listed below expansion of CRISPR-1 was detected after one round of infectivity.

Gene deleted	Strain name. Details in Table S2B	Function and example reference
recA	IIB971 & IIB1013	Homologous recombination strand exchange recombinase. [18]
recE	IIB1031	DNA exonuclease. [19]
recF	IIB993	RecFOR recombination. [20, 21]
recJ	IIB992	5' to 3' ssDNA nuclease. [18]
recQ	IIB996	Helicase/translocase for genome stability. [22]
ruvABC	IIB1006	Helicase-resolvase for homologous recombination (RuvABC resolvasome) [23, 24].
rep	IIB1071	Helicase/translocase for replication re-start by primosome assembly [25].
priB	IIB1081	Primosome assembly protein of PriA-PriB pathway for replication restart [26].
priC	IIB1069	Primosome assembly protein of PriA-PriC pathway for replication restart [27].
rnhA	IIB1008	RNase H. Degrades RNA in RNA-DNA hybrids and R-loops.
htpG	IIB990	Chaperone for Cas3 [28].
sbcD	IIB1024	SbcCD hairpin exo- and endonuclease. [29].
hns	IIB994	DNA binding, packaging and regulatory protein.

PCR primers for CRISPR expansion assays

Spacer acquisition was detected as expansion of CRISPR locus, by PCR amplification of genomic DNA or bacterial culture using the primers listed below.

(a) For primed acquisition:

CRISPR-1F: 5’-GAGATGCAGGCCATCGGA

CRISPR-1R: 5’-GCGACCGCTCAGAAATTCCAGACCCGATCCAAA

(b) For naïve acquisition:

CRISPR-1F-EB: 5’-GTACCTCCGCGCTTACGAGG, and CRISPR-1R

(c) For CRISPR-2 in primed or naïve acquisition:

EC_II F 5'-AACATAATGGATGTGTTGTTTGTG-3'

EC_II R 5'-GAAATGCTGGTGAGCGTTAATG-3'

Table S5A. Plating efficiency of λ*vir* on different strains grown to stationary phase. Infectivity of phage was not significantly reduced by gene deletions that abolished primed spacer acquisition.

Strain	Mean efficiency of plating (t=3) with standard deviation
IIB969 (*wt*)	2.86×10^{10} +/- 4.0
IIB998 (Δ*recG*)	3.97×10^{10} +/- 1.1
IIB1012 (Δ*polA*)	2.83×10^{10}+/- 5.1
IIB1020 (Δ*priA*)	1.63×10^{10}+/- 5.5

Table S5B. Comparison of strain growth, measured as optical density (OD) of growing cultures after reaching OD 0.6, the point at which phage λ*vir* was added.

Strain	OD_{600} measured at times after reaching OD for phage		
	0 (phage added)	60 min.	120 min.
IIB969 (*wt*)	0.58	1.26	1.76
IIB998 (Δ*recG*)	0.58	1.37	1.78
IIB1012 (Δ*polA*)	0.59	1.27	1.73
IIB1020 (Δ*priA*)	0.58	1.10	1.42

Mutational analysis of *E. coli* Cas1 Arg-84, Arg-95, Arg-123 and Arg-138

Supplementary Figure S3A details analysis of Cas1 to unearth potential DNA binding amino acid residues, leading to our selection of four arginine residues for experimental analysis in this work. In summary, atomic co-ordinates of *E. coli* Cas1 protein in complex with Cas2 (PDB 4P6I) were interrogated using MOLE to determine positions of potentially interesting unreported amino acid residues in Cas1 that might form channels for DNA binding. Two key areas were identified: the α-helical Cas1 C-terminal region (96-305) that contains the enzyme catalytic site, and an area (1-96) proximal to the linker region between Cas1 monomers. These two regions gave a total of four possible DNA binding channels (Fig S3A). ClustalW alignments comparing Cas1 from *E. coli* and closely related species (*Salmonella enterica*, *Shigella flexneri*, *Yersinia pestis*, *Photorhabdus asymbiotica* and *Pseudomonas aeruginosa*) refined this list to 24 lysine, arginine or histidine residues that were invariant or strongly conserved, several of which had been described for DNA binding or Cas1-Cas2 complex formation previously in Babu et al., or Nunez *et* al (2014), as indicated in Figure S3A.

Of the 24 *E. coli* Cas1 residues identified by MOLE and Clustal, we selected four for mutagenesis that had not been previously studied (Figure S3B-D): (i) R123 and R138, each adjacent to the metal binding pocket on separate α-helices facing toward the active site. (ii) R84 and R95 located, respectively, within an N-terminal β-strand orientated towards the active site, and in a short connector region between the β-strand and a larger α-helix facing away from R84. Each was mutagenized to glycine within pEB525 and pEB628 (Table S3) using the New England Biolabs Base-Changer Q5 system with primers listed below. Mutations were verified in each case by DNA sequencing:

Cas1 DNAbind Arg84_F 5'-TGGAGGTGCGGGTTCAGATAAG
Cas1 DNAbind Arg84_R 5'-GGCTGACCAGAAGCATAAAC
Cas1 DNAbind Arg95_F 5'-AGCGGGCGTTGGTGTTTATGC
Cas1 DNAbind Arg95_R 5'-TCCCCCACCCATACCAAC
Cas1 DNAbind Arg123_F 5'-GTTTGAACTTGGGTTTGGAGAAC
Cas1 DNAbind Arg123_R 5'-ATTTTACGTACGACCTTCAG
Cas1 DNAbind Arg138_F 5'-AGAGCAACTCGGAGGTATAGAAGGCAGTCGCG
Cas1 DNAbind Arg138_R 5'-ACGGAGCGCCGGGCAGGC

Arginine mutant derivatives of pEB628 were each tested for spacer acquisition in naïve and primed assays as described in the main results.

DNA oligonucleotides and *Chi* substrates for Cas1-Cas2 assays *in vitro*

Oligonucleotide sequences and structures that were used to generate substrates for Cas1 EMSA and catalytic analysis are summarized below and illustrated in Figure S5A.

Fork-1: Nucleotides forming this fork are given in Figure S5A, derived by annealing strands 1, 2 and 3 as indicated in the Figure.

Fork 1a: Nucleotides forming this fork are given in Figure S5A. As Fork-1 but with addition of strand 4 to anneal to strand 1 giving a 4 nt ssDNA gap at the fork branch point.

Fork 1b: As Fork-1 but with addition of strand 5 to anneal to strand 1 giving a 2 nt ssDNA gap.

Fork-2: Strand 3 is not included, and addition of strand 6 generates a fork of opposite polarity ssDNA to fork-1.

Fork-3: As fork-1 but annealing strand 6 to strand 1 giving a fully base-paired fork.

Holliday junction:

Strand 1 and Strand 2 shown in Figure S5 were annealed to two additional 50 nucleotide strands to give a fully base-paired Holliday junction:

Strand 7: 5'-TGCCGAATTCTACCAGTGCCAGTGATGGACATCTTTGCCCACGTTGACCC

Strand 8: 5'-TGGGTCAACGTGGGCAAAGATGTCCTAGCAATGTAATCGTCTATGACGTT

Holliday junction (*Chi*) and fork (*Chi*^{Sma}) substrates

These were generated from supercoiled plasmid according to the method detailed in [30, 31].

SUPPLEMENTARY REFERENCES

1. Datsenko, K.A. and B.L. Wanner, *One-step inactivation of chromosomal genes in Escherichia coli K-12 using PCR products.* Proc. Natl. Acad. Sci. U S A, 2000. **97**(12): p. 6640-5.
2. Pougach, K., et al., *Transcription, processing and function of CRISPR cassettes in Escherichia coli.* Mol. Microbiol., 2010. **77**(6): p. 1367-79.
3. Datsenko, K.A., et al., *Molecular memory of prior infections activates the CRISPR/Cas adaptive bacterial immunity system.* Nat. Commun., 2012. **3**: p. 945.
4. Howard, J.A., et al., *Helicase dissociation and annealing of RNA-DNA hybrids by Escherichia coli Cas3 protein.* Biochem. J, 2011. **439**(1): p. 85-95.
5. Sandler, S.J., H.S. Samra, and A.J. Clark, *Differential suppression of priA2::kan phenotypes in Escherichia coli K-12 by mutations in priA, lexA, and dnaC.* Genetics, 1996. **143**: p. 5-13.
6. Mahdi, A.A., G.S. Briggs, and R.G. Lloyd, *Modulation of DNA damage tolerance in Escherichia coli recG and ruv strains by mutations affecting PriB, the ribosome and RNA polymerase.* Mol. Microbiol., 2012. **86**(3): p. 675-91.
7. Jaktaji, R.P. and R.G. Lloyd, *PriA supports two distinct pathways for replication restart in UV-irradiated Escherichia coli cells.* Mol. Microbiol., 2003. **47**(4): p. 1091-1100.
8. Rudolph, C.J., et al., *RecG protein and single-strand DNA exonucleases avoid cell lethality associated with PriA helicase activity in Escherichia coli.* Genetics, 2010. **186**(2): p. 473-92.
9. Zhang, J., et al., *Promoting and avoiding recombination: contrasting activities of the Escherichia coli RuvABC Holliday junction resolvase and RecG DNA translocase.* Genetics, 2010. **185**(1): p. 23-37.
10. Stockum, A., R.G. Lloyd, and C.J. Rudolph, *On the viability of Escherichia coli cells lacking DNA topoisomerase I.* BMC Microbiol., 2012. **12**: p. 26.
11. Lloyd, R.G., C. Buckman, and F.E. Benson, *Genetic analysis of conjugational recombination in Escherichia coli K-12 strains deficient in RecBCD enzyme.* J. Gen. Microbiol., 1987. **133**: p. 2531-2538.
12. Baba, T., et al., *Construction of Escherichia coli K-12 in-frame, single-gene knockout mutants: the Keio collection.* Mol. Syst. Biol., 2006. **2**: p. 2006 0008.
13. Ivancic-Bace, I., E. Salaj-Smic, and K. Brcic-Kostic, *Effects of recJ, recQ, and recFOR mutations on recombination in nuclease-deficient recB recD double mutants of Escherichia coli.* J. Bacteriol., 2005. **187**(4): p. 1350-6.
14. Dermic, D. and Z. Trgovcevic, *Specific effects of a recB mutation on the HfrH strain of Escherichia coli.* J. Bacteriol., 1999. **181**(4): p. 1334-7.
15. Zahradka, K., et al., *Roles of ExoI and SbcCD nucleases in "reckless" DNA degradation in recA mutants of Escherichia coli.* J. Bacteriol., 2009. **191**(5): p. 1677-87.
16. Upton, A.L., et al., *Cellular location and activity of Escherichia coli RecG proteins shed light on the function of its structurally unresolved C-terminus.* Nucleic Acids Research, 2014. **42**(9): p. 5702-14.
17. Zavitz, K.H. and K.J. Marians, *ATPase-deficient mutants of the Escherichia coli DNA replication protein PriA are capable of catalyzing the assembly of active primosomes.* J. Biol. Chem., 1992. **267**(10): p. 6933-6940.
18. Kowalczykowski, S.C., et al., *Biochemistry of homologous recombination in Escherichia coli.* Microbiol. Rev., 1994. **58**: p. 401-465.
19. Kolodner, R., S.D. Hall, and C. Luisi-DeLuca, *Homologous pairing proteins encoded by the Escherichia coli recE and recT genes.* Mol. Microbiol., 1994. **11**: p. 23-30.
20. Michel, B., et al., *Recombination proteins and rescue of arrested replication forks.* DNA Repair (Amst), 2007. **6**(7): p. 967-80.
21. Morimatsu, K. and S.C. Kowalczykowski, *RecFOR Proteins Load RecA Protein onto Gapped DNA to Accelerate DNA Strand Exchange. A Universal Step of Recombinational Repair.* Mol. Cell, 2003. **11**(5): p. 1337-47.
22. Morimatsu, K. and S.C. Kowalczykowski, *RecQ helicase and RecJ nuclease provide complementary functions to resect DNA for homologous recombination.* Proc. Natl. Acad. Sci. U. S. A., 2014. **111**(48): p. E5133-42.

23. Seigneur, M., et al., *RuvAB acts at arrested replication forks.* Cell, 1998. **95**(3): p. 419-30.
24. Whitby, M.C., et al., *Interactions between RuvA and RuvC at Holliday junctions: inhibition of junction cleavage and formation of a RuvA-RuvC-DNA complex.* J. Mol. Biol., 1996. **264**: p. 878-890.
25. Guy, C.P., et al., *Rep provides a second motor at the replisome to promote duplication of protein-bound DNA.* Mol. Cell, 2009. **36**(4): p. 654-66.
26. Liu, J., P. Nurse, and K.J. Marians, *The ordered assesmbly of the øX174-type primosome III. PriB facilitates complex formation between PriA and DnaT.* J. Biol. Chem., 1996. **271**: p. 15656-15661.
27. Heller, R.C. and K.J. Marians, *Unwinding of the nascent lagging strand by Rep and PriA enables direct restart of stalled replication forks.* J. Biol. Chem., 2005.
28. Yosef, I., et al., *High-temperature protein G is essential for activity of the Escherichia coli clustered regularly interspaced short palindromic repeats (CRISPR)/Cas system.* Proc. Natl. Acad. Sci. U. S. A., 2011. **108**(50): p. 20136-41.
29. Darmon, E. and D.R. Leach, *Bacterial genome instability.* Microbiol. Mol. Biol. Rev., 2014. **78**(1): p. 1-39.
30. Zerbib, D., et al., *Effect of DNA topology on Holliday junction resolution by Escherichia coli RuvC and bacteriophage T7 endonuclease I.* J. Mol. Biol., 1997. **270**(5): p. 663-73.
31. McGlynn, P. and R.G. Lloyd, *Modulation of RNA polymerase by (p)ppGpp reveals a RecG-dependent mechanism for replication fork progression.* Cell, 2000. **101**(1): p. 35-45.
32. Babu, M., et al., *A dual function of the CRISPR-Cas system in bacterial antivirus immunity and DNA repair.* Mol. Microbiol., 2011. **79**(2): p. 484-502.
33. Nunez, J.K., et al., *Cas1-Cas2 complex formation mediates spacer acquisition during CRISPR-Cas adaptive immunity.* Nat. Struct. Mol. Biol., 2014. **21**(6): p. 528-34.

Figure S1

A.

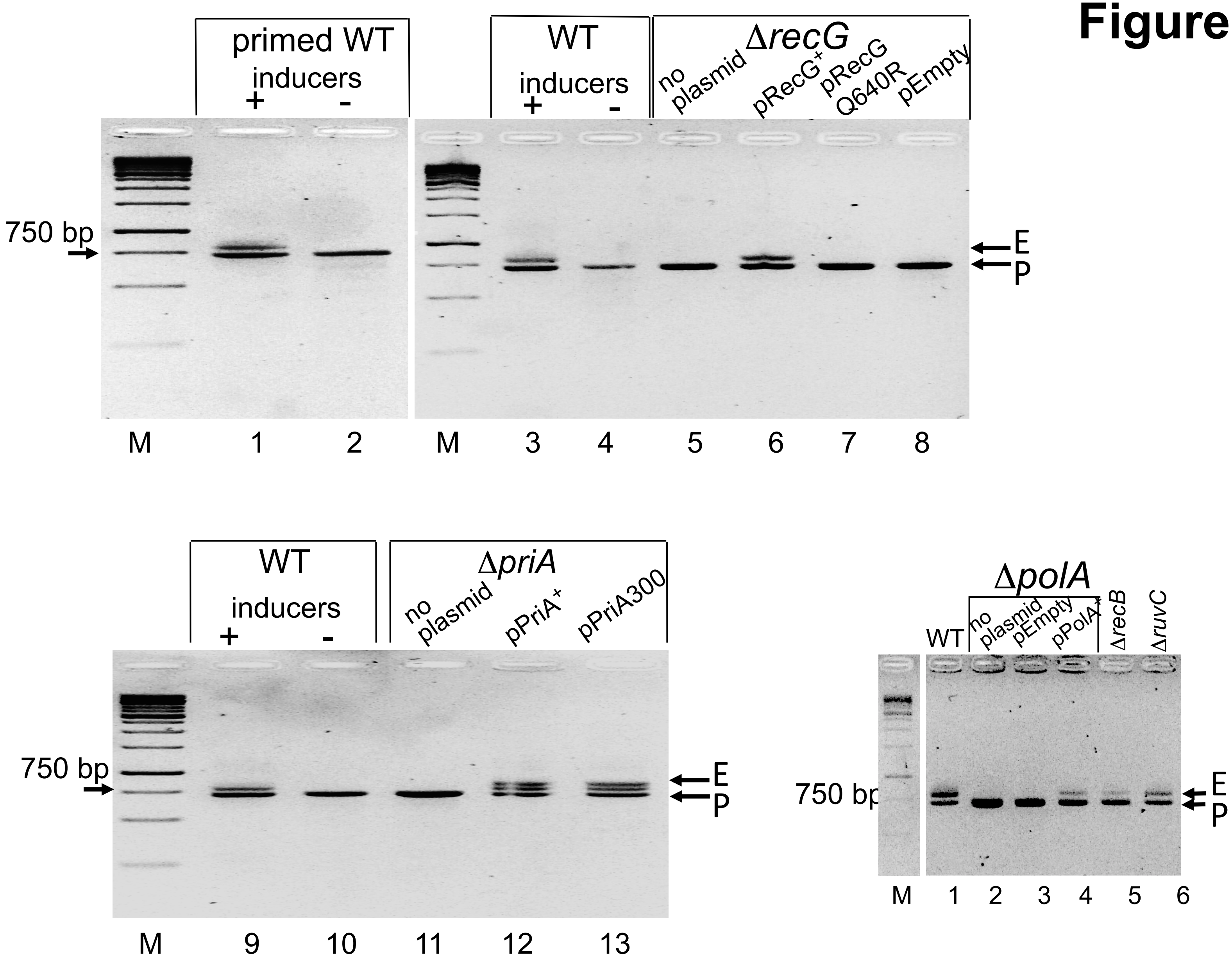

Figure S1. (A). Untrimmed agarose gel images of the cropped gel images presented in Figure 1B and Figure 1C.

Figure S1

B.

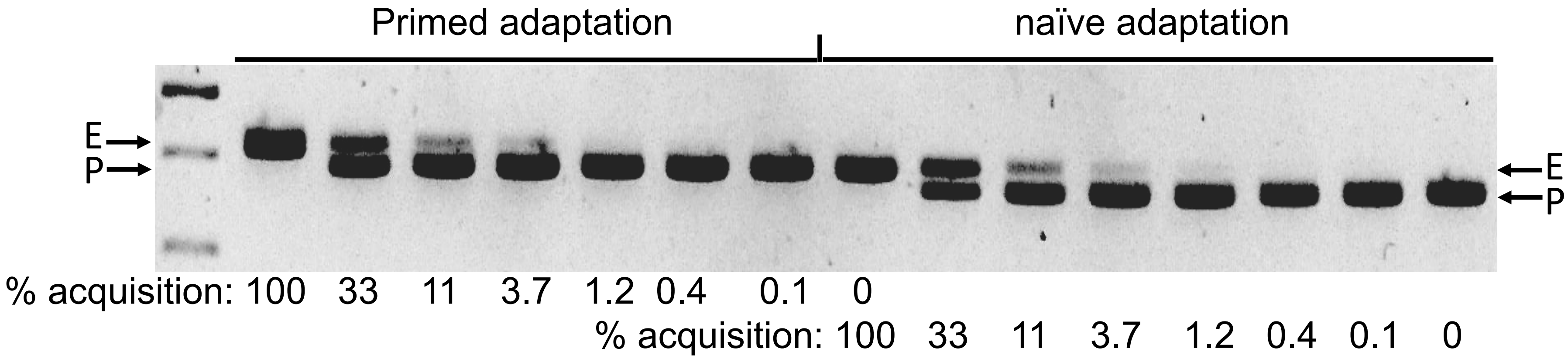

Figure S1 (B). Agarose gel showing progressive spacer acquisition in primed or naïve adaptation assays. Below each lane is noted DNA present as expanded (E) CRISPR as a % of the lane total of E + parental (P) CRISPR.

Figure S2

A.

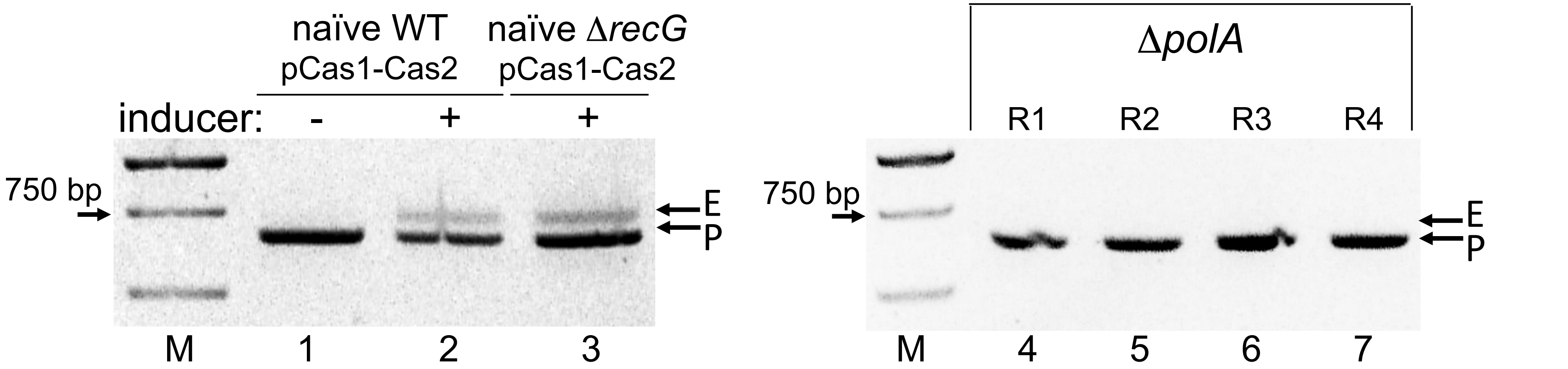

B.

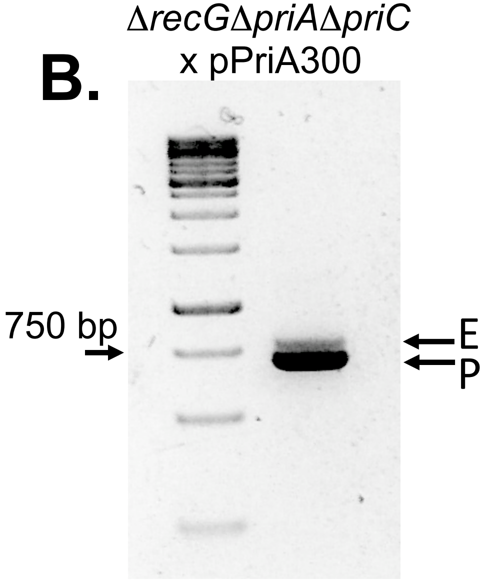

Figure S2. (A). Agarose gels of DNA from PCR reactions to detect expansion (E) of CRISPR from its parental length (P) in naïve adaptation in induced and uninduced wild type cells (lanes 1 and 2) and when RecG was eliminated (lane 3). Additionally, lanes 4-7 show the inability to detect any CRISPR-1 expansion by naïve adaptation in cells lacking PolA, even after multiple rounds of infectivity (R1-R4). **(B).** Agarose gels showing spacer acquisition in the strain lacking *recG*, *priA* and *priC* and expressing PriA300 from a plasmid as indicted.

Figure S3

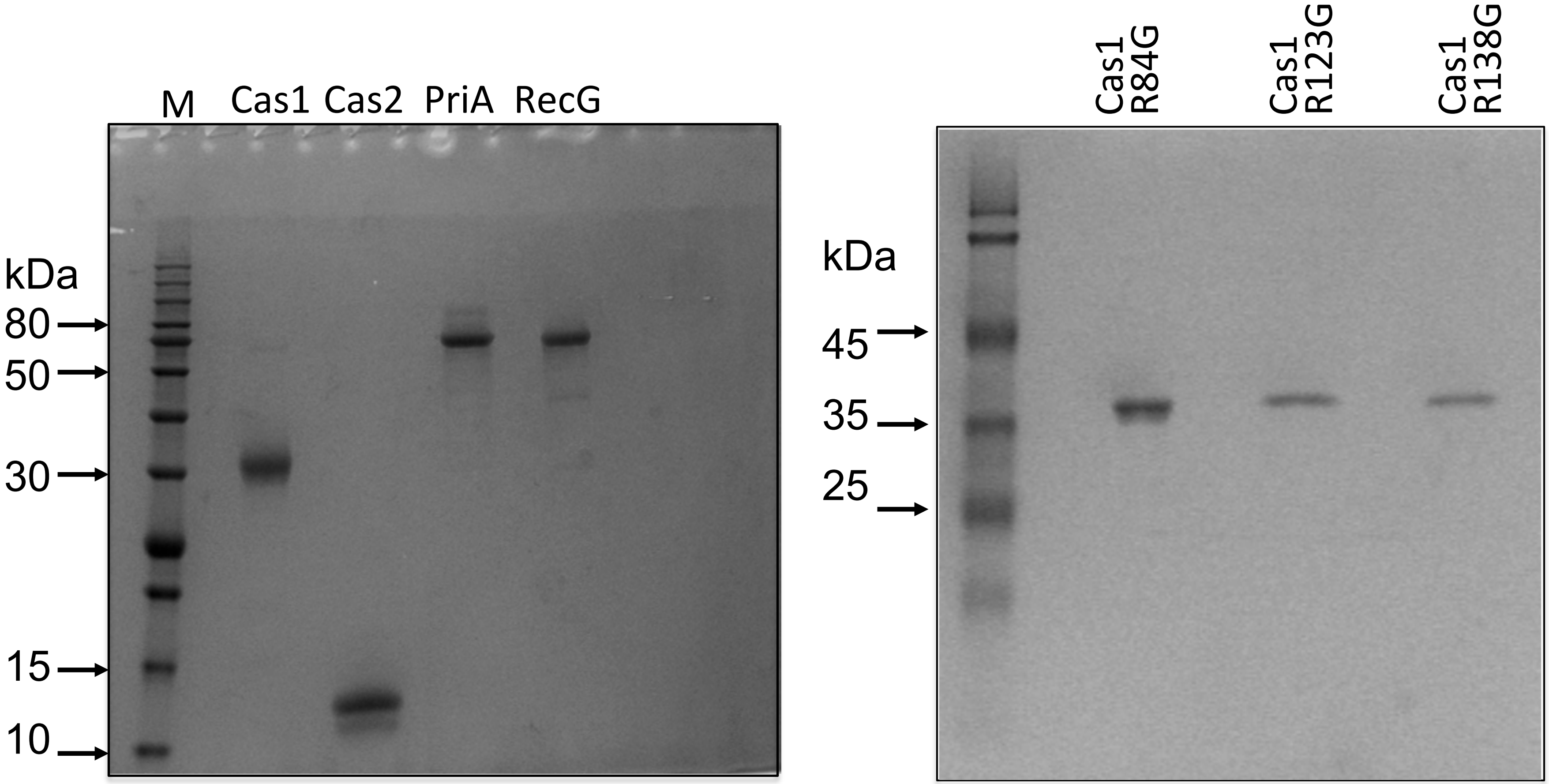

Figure S3. Coomassie stained SDS-PAGE gels showing loadings of purified proteins (1-2 μg) used in this study as annotated above each panel.

Figure S4

Figure S4. Structures and nucleotide sequences of forked DNA used in this study. The position of ^{32}P end labeling is marked by * in each case. Arrows indicate positions of DNA strand cleavage by Cas1 (see also Figure 3F).

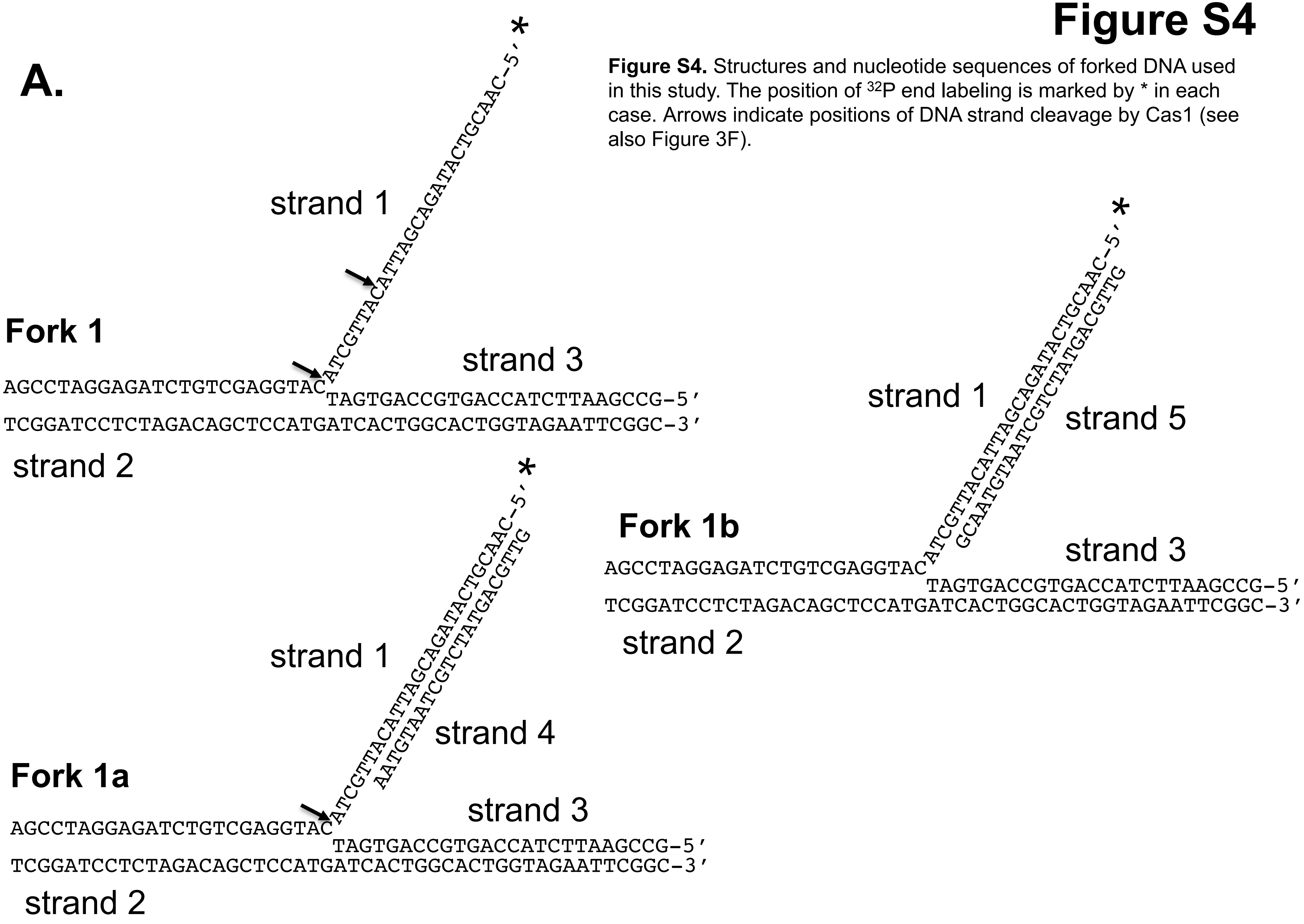

Figure S4

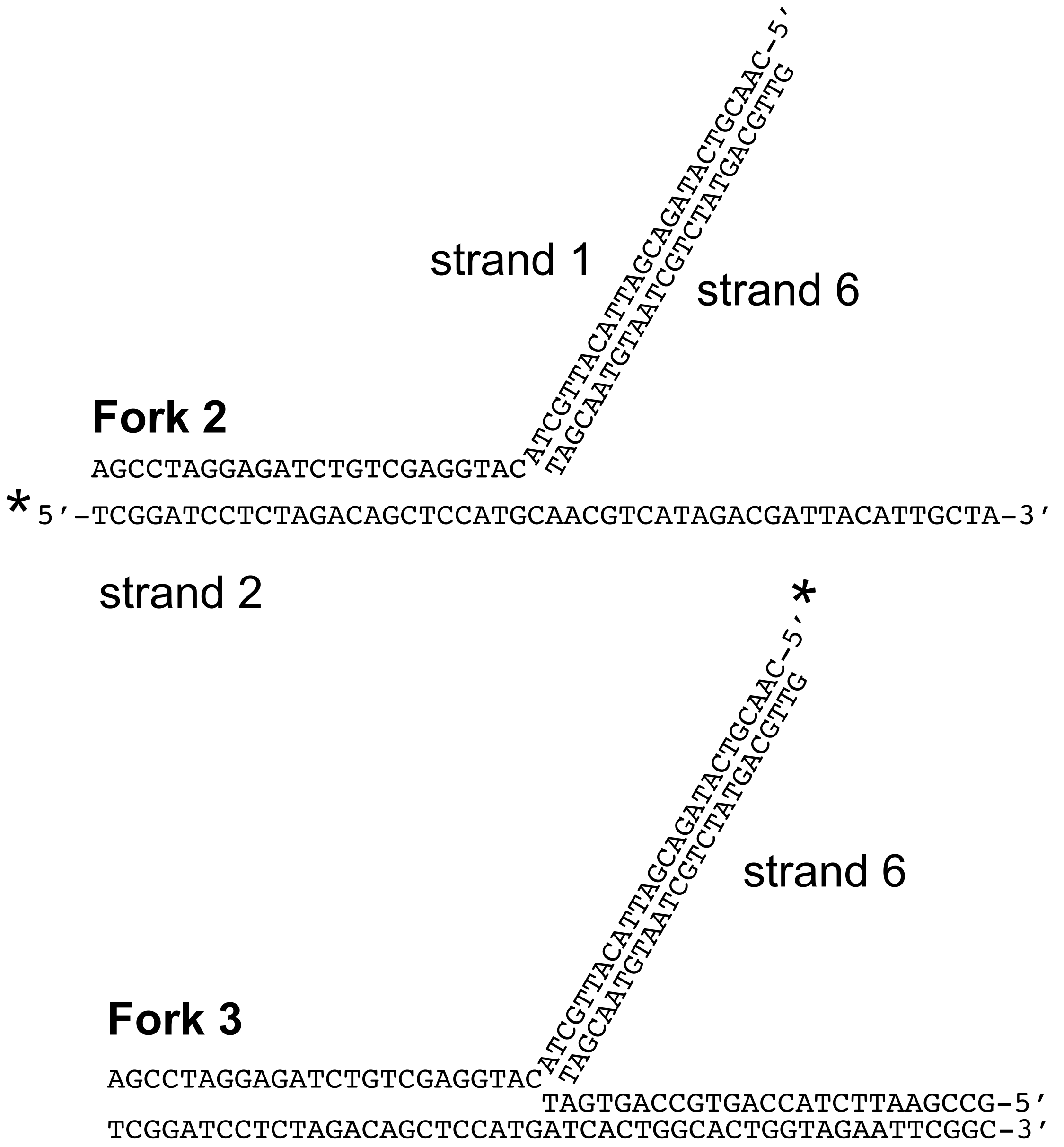
strand 1
ATCGTTACATTAGCAGATACTGCAAC-5'
TAGCAATGTAATCGTCTATGACGTTG
strand 6
Fork 2
AGCCTAGGAGATCTGTCGAGGTAC
*5'-TCGGATCCTCTAGACAGCTCCATGCAACGTCATAGACGATTACATTGCTA-3'
strand 2
*
ATCGTTACATTAGCAGATACTGCAAC-5'
TAGCAATGTAATCGTCTATGACGTTG
strand 6
Fork 3
AGCCTAGGAGATCTGTCGAGGTAC
TAGTGACCGTGACCATCTTAAGCCG-5'
TCGGATCCTCTAGACAGCTCCATGATCACTGGCACTGGTAGAATTCGGC-3'

Figure S5

A

	Analysis	*E. coli* Cas1 Residues					
1	MOLE 2.0 Study	K12	R14				
	ClustalW Alignment	K12.	R14*	K37.	H43:		H62:
	Babu et al., 2011			K37‡		R59‡	
	**Analysis**	***E. coli* Cas1 Residues**					
2	MOLE 2.0 Study	R66	R84	R95	R112	R123	R138
	ClustalW Alignment	R66:			R112*	R123*	R138*
	Babu et al., 2011		R84‡		R112†	R123†	R138†
	**Analysis**	***E. coli* Cas1 Residues**					
3	MOLE 2.0 Study	R146	R163			H208	
	ClustalW Alignment			K169.	K172:	H208:	K211:
	Babu et al., 2011	R146†	R163†				K211†
	**Analysis**	***E. coli* Cas1 Residues**					
4	MOLE 2.0 Study	K224		R245	R248	R252	
	ClustalW Alignment	K224.	K231:			R252:	K259
	Babu et al., 2011	K224†		R245‡	R248‡	R252‡	

B

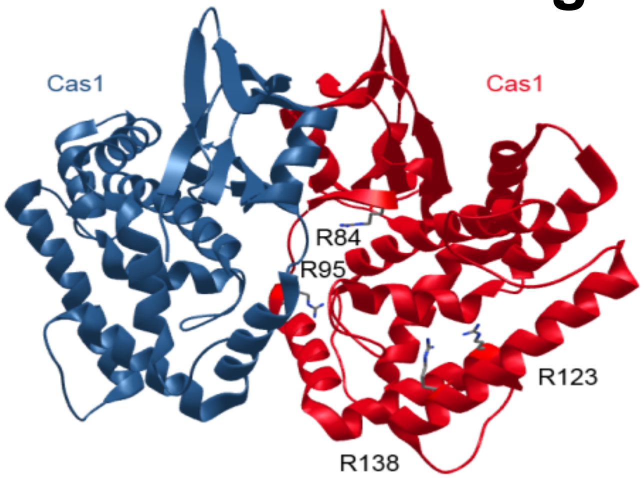

C

D

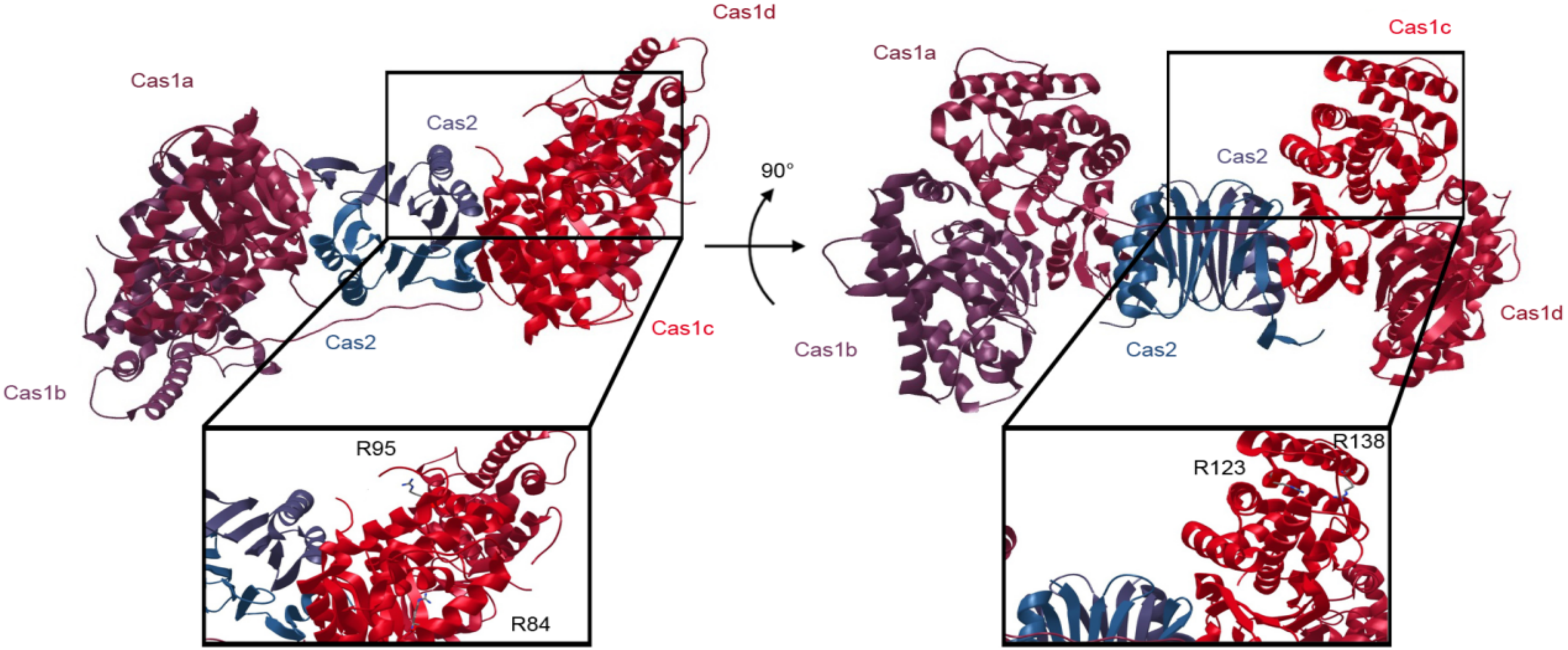

Figure S5

E.

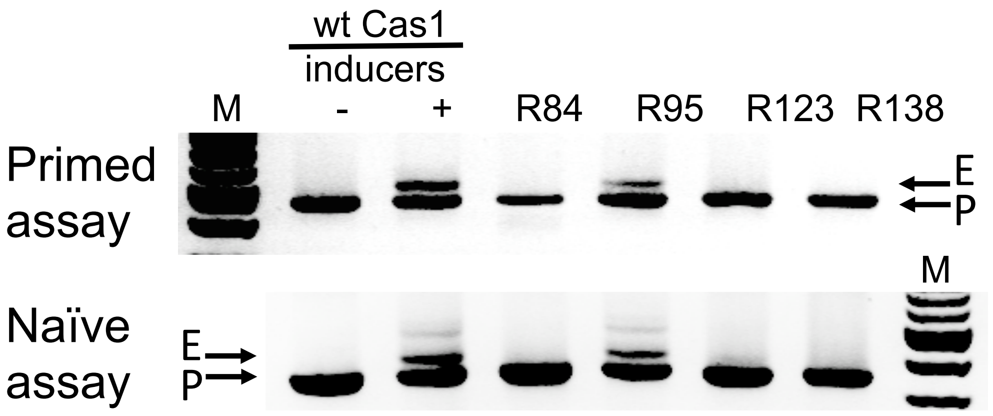

F.

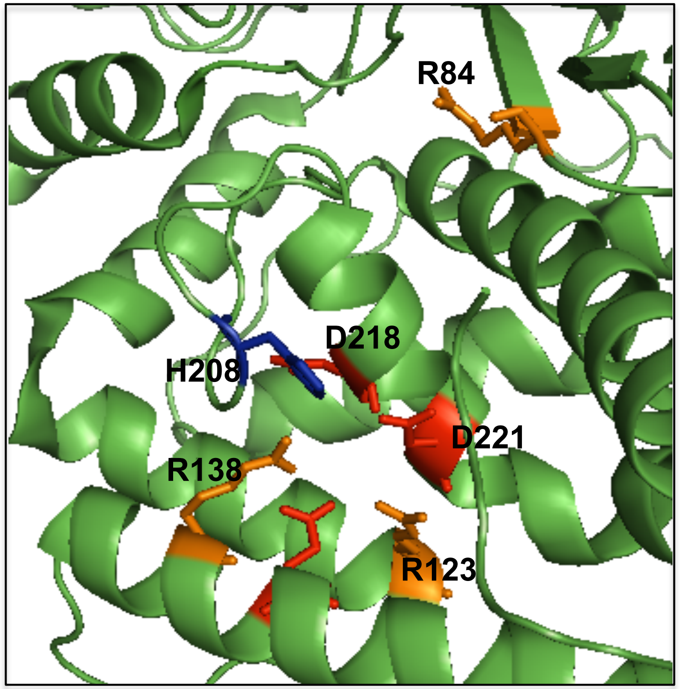

Figure S5. Analysis of *E. coli* Cas1-DNA binding residues to identify four arginine residues reported in this study. (A) MOLE identified four potential DNA binding channels present in Cas1 from PDB accession 4P6I, as labeled next to the table. Lysine, arginine and histidine residues were selected for further interrogation. ClustalW alignment of Cas1 amino acid sequences from several bacterial species identified which of these residues are invariable (*) or conserved (:). Residues implicated in DNA binding by Babu et al., 2011 were compared, where (†) represents residues proximal to the active site and (‡) represents residues distal from the active site. **(B)** Cas1 dimer structure highlighting on a Cas1 monomer the four arginine residues chosen for mutation. **(C)** Cas1-Cas2 complex highlighting the position and orientation of the R84 and R95 residues within Cas1c monomer. **(D)** Cas1-Cas2 complex rotated 90° from (C) to display amino acids R123 and R138 within Cas1c monomer. **(E).** Analysis of spacer acquisition into *E. coli* CRISPR in cells expressing Cas1 arginine mutants, assessed on agarose gels after PCR reactions. Both primed and naïve adaptation assays used inducible expression of Cas1 from plasmids (wild type from pEB628 or pSJW5-8, Table S3) as indicated above the panel. Primed adaptation was analyzed as described in the main methods and results except that the strain used (IIB969, Table S2) contained an additional deletion of chromosomal *cas1* (strain EB358, Table S2) so that only plasmid encoded Cas1 protein was present. **(F).** Catalytic residues of *E. coli* published in previous work (His-208, Asp-218 and Asp-221) highlighted with positions of the Cas1 residues that were newly identified in this work as being required for spacer acquisition: Arg-84, Arg-123 and Arg-138. The image was created on PyMol from PDB accession 4P6I.

Figure S6

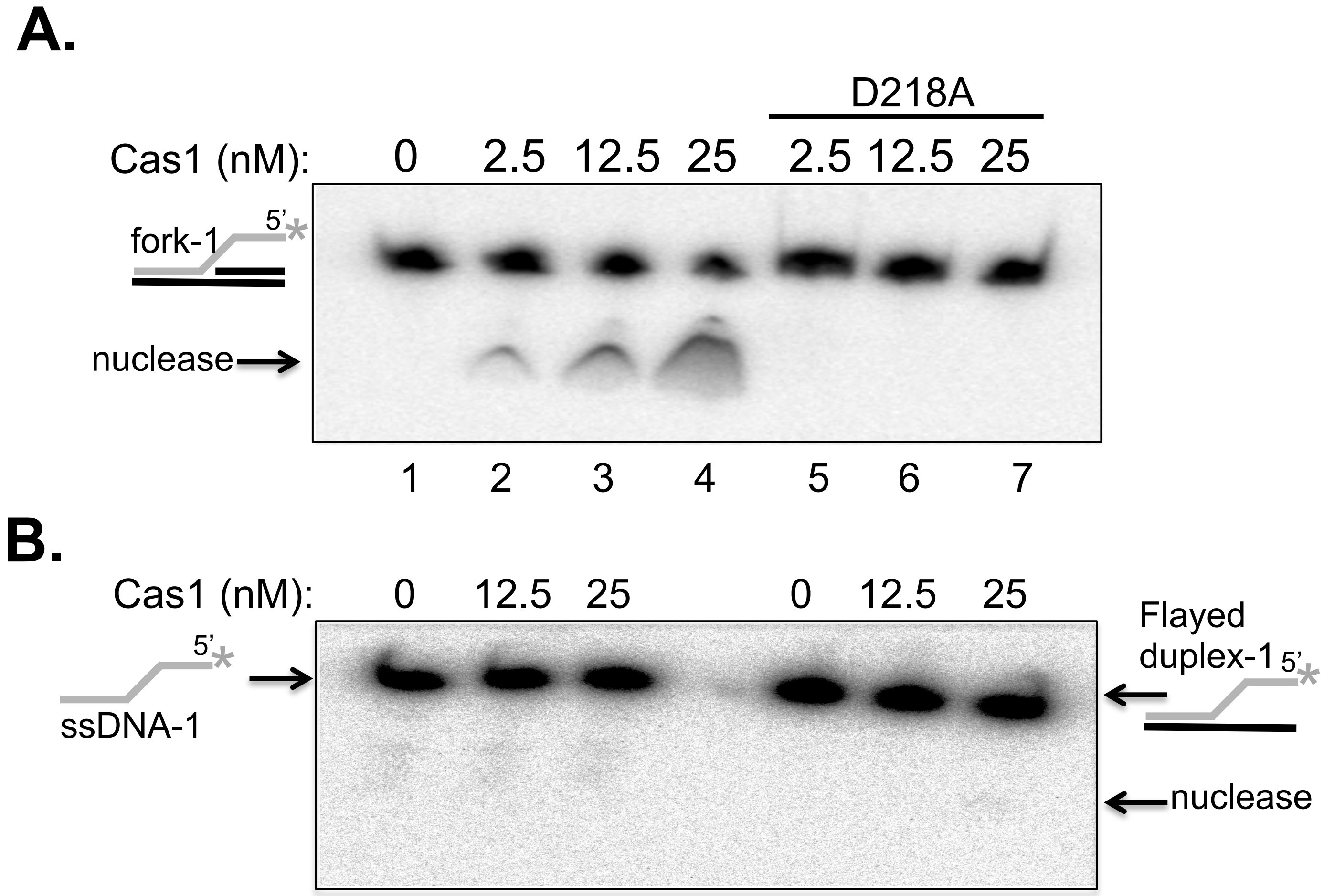

Figure S6 (A). Urea DNA denaturing gel to detect nicking of the 5'-end labeled strand (*) of fork-1 DNA (6 nM) by Cas1 or D218A Cas1 as indicated (0, 2.5, 12.5 and 25 nM). **(B).** Urea gel showing activity of Cas1 (0, 2.5, 25 nM), as indicated, when mixed in cleavage assays with 6 nM of ssDNA or a flayed duplex in each case ^{32}P end-labeled on the same DNA strand as was cleaved in Fork-1.

Figure S6

C.

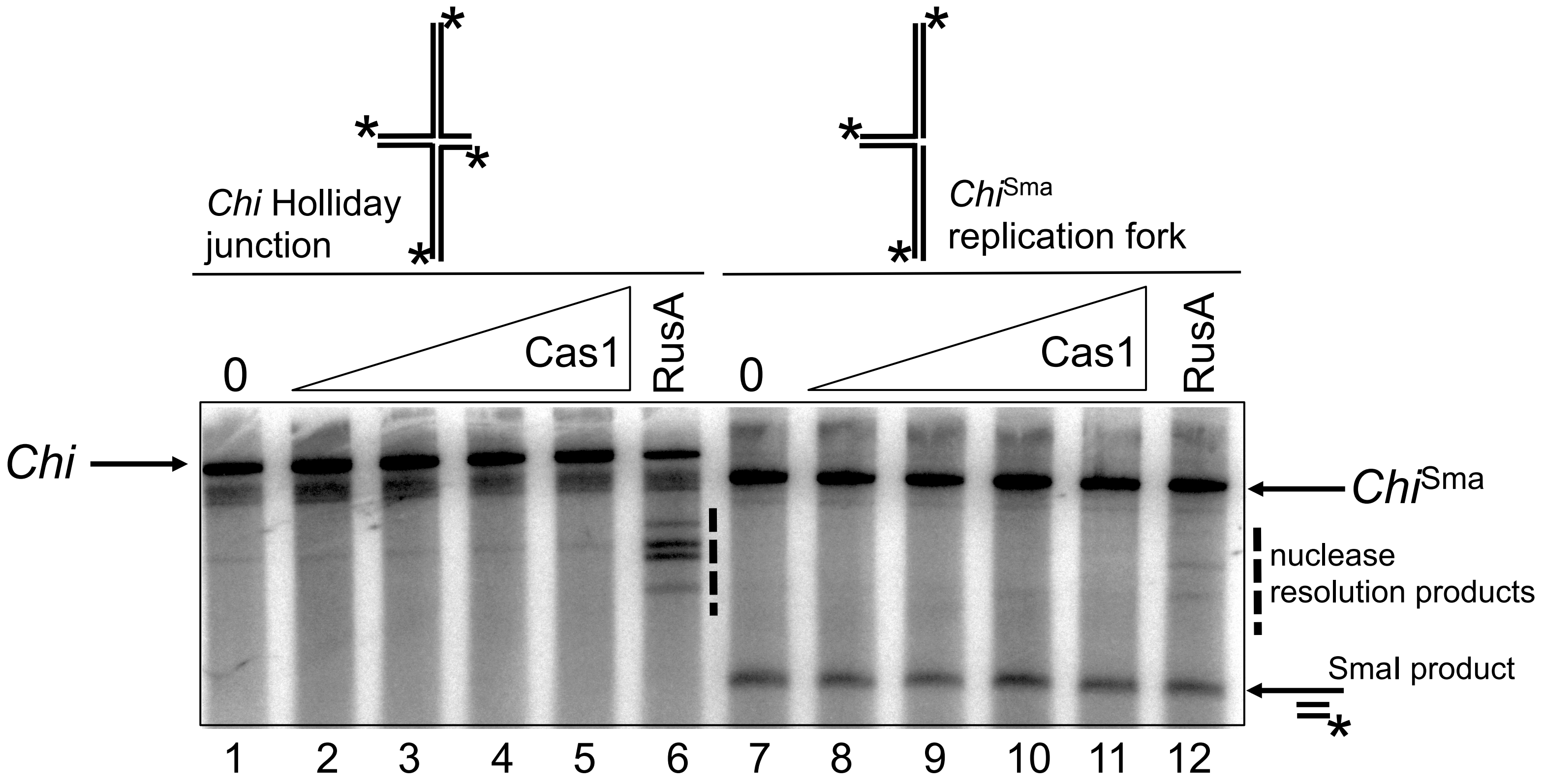

(C). Agarose gel showing outcomes of titrating Cas1 (0, 2.5, 25, 100 and 250nM) into 0.1 ng of large plasmid derived ^{32}P 5' end labeled (*) plasmid derived substrates *Chi* (lanes 1-6) or *Chi*^{Sma} (lanes 7-12) that mimic a Holliday junction or replication fork, respectively. RusA (50 nM), which cuts Holliday junctions and has weak activity on forks (indicated by the dotted line), was used as a positive control.

Figure S6

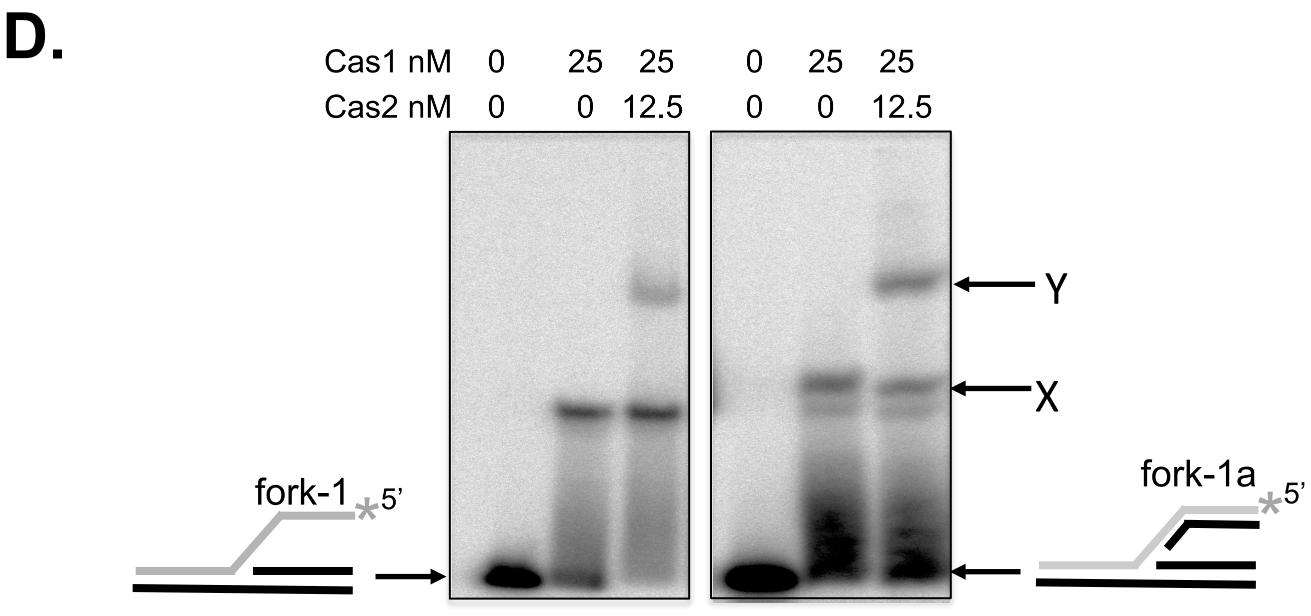

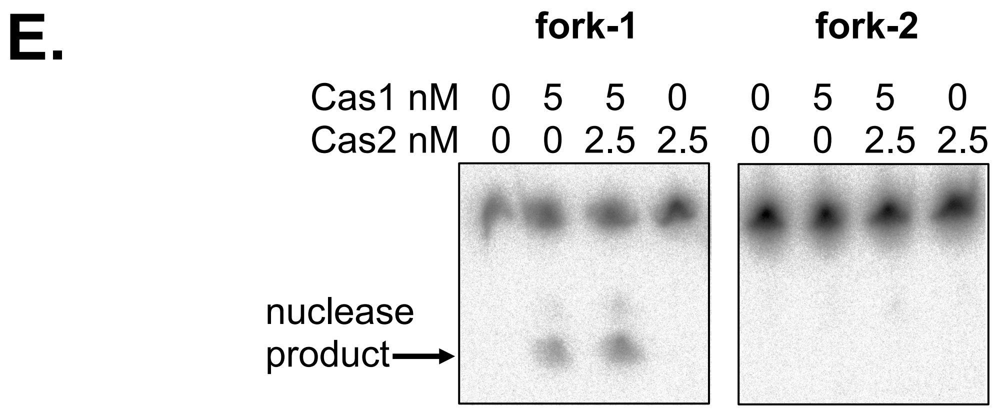

Figure S6 (D). EMSAs showing binding of Cas1 (25 nM) and Cas1 with Cas2 (12.5 nM), as indicated, to 6 nM of fork-1 or fork-1a. **(E).** Urea DNA denaturing gels of Cas1 (25 nM) cutting either fork-1 or fork-2 (both 6 nM) after pre-incubation with Cas2.

Figure S7

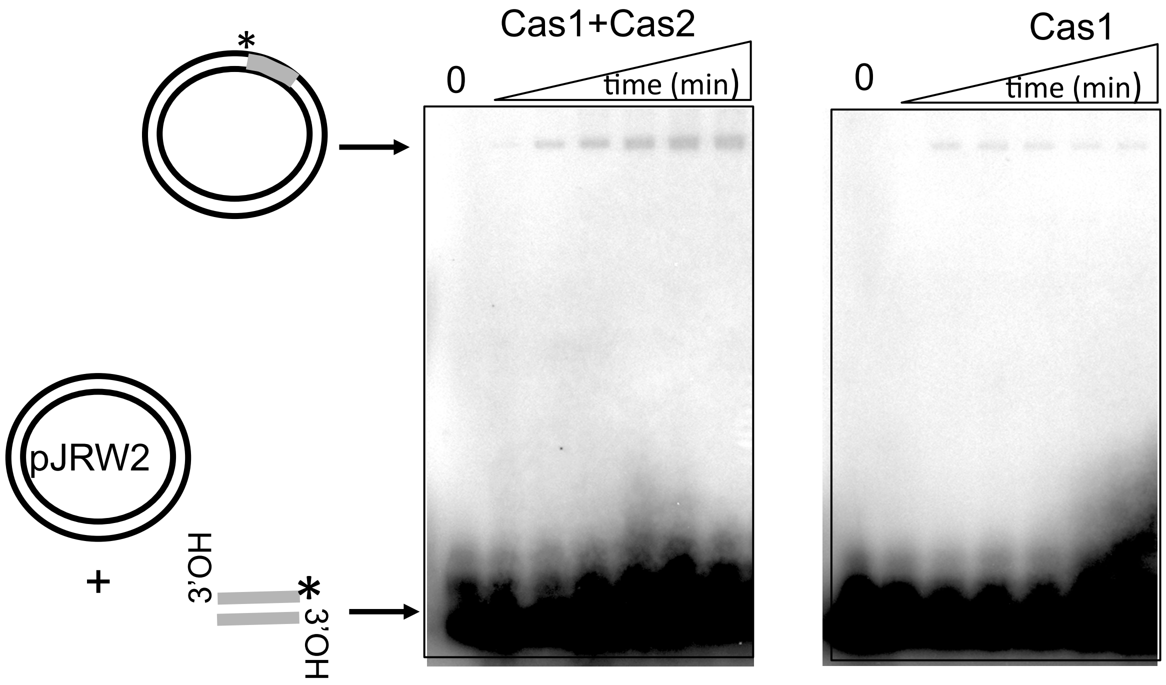

Figure S7. Agarose gels to detect integration of an end labeled (*) 35 base pair duplex DNA into plasmid pJRW2 catalysed by Cas1 (100 nM) or Cas1 and Cas2 (100 and 50 nM respectively) incubated for 5, 10, 15, 20, 25 and 30 minutes).
